# Structural basis of the recognition of adeno-associated virus by the neurological system-related receptor carbonic anhydrase IV

**DOI:** 10.1371/journal.ppat.1011953

**Published:** 2024-02-05

**Authors:** Ran Zhang, Yixiao Liu, Fengxi Yu, Guangxue Xu, Lili Li, Baobin Li, Zhiyong Lou

**Affiliations:** 1 Jinshan Hospital, Institute for Translational Brain Research, Fudan University, Shanghai, China; 2 MOE Key Laboratory of Protein Science, School of Medicine, Tsinghua University, Beijing, China; 3 School of Life Sciences, Tsinghua University, Beijing, China; 4 Department of Pathology, Stanford University School of Medicine, Stanford, California, United States of America; 5 Beijing Institute of Biological Products Company Limited, Beijing, China; 6 Department of Anesthesiology, Zhongshan Hospital, Institute for Translational Brain Research, State Key Laboratory of Medical Neurobiology, MOE Frontiers Center for Brain Science, Fudan University, Shanghai, China; Cardiff University, UNITED KINGDOM

## Abstract

Carbonic anhydrase IV (Car4) is a newly identified receptor that allows adeno-associated virus (AAV) 9P31 to cross the blood-brain barrier and achieve efficient infection in the central nervous system (CNS) in mouse models. However, the molecular mechanism by which engineered AAV capsids with 7-mer insertion in the variable region (VR) VIII recognize these novel cellular receptors is unknown. Here we report the cryo-EM structures of AAV9P31 and its complex with *Mus musculus* Car4 at atomic resolution by utilizing the block-based reconstruction (BBR) method. The structures demonstrated that Car4 binds to the protrusions at 3-fold axes of the capsid. The inserted 7-mer extends into a hydrophobic region near the catalytic center of Car4 to form stable interactions. Mutagenesis studies also identified the key residues in Car4 responsible for the AAV9P31 interaction. These findings provide new insights into the novel receptor recognition mechanism of AAV generated by directed evolution and highlight the application of the BBR method to studying the virus-receptor molecular mechanism.

## Author summary

Recombinant adeno-associated viruses (AAVs) are promising vectors for gene therapy, especially for metabolic, neurological and hematological diseases. Among them, AAV9P31, which was designed based on the AAV9 backbone, is a highly effective candidate for gene delivery in the central nervous system. To optimize this vector, it is crucial to understand how AAV9P31 recognizes its receptor carbonic anhydrase IV (Car4) and crosses the blood-brain barrier. In this study, we determined the atomic structures of AAV9P31 in its native state and in complex with Car4, revealing the molecular details of virus-receptor interactions. Moreover, we applied the block-based reconstruction algorithm to capture the asymmetric binding of the receptor on the icosahedral virus, which is important for studying recombinant AAV targeting neurological receptors.

## Introduction

Adeno-associated viruses (AAVs) are a type of single-stranded DNA virus within the *Parvoviridae* family [1]. Because of the broad differences in their tissue tropism and the absence of pathology, AAVs considered promising vectors for therapeutic gene delivery [1,2]. To date, Luxturna, Zolgensma, Elevidys and Roctavian have been approved by the FDA for the treatment of hereditary retinal dystrophy, spinal muscular atrophy, Duchenne muscular dystrophy and haemophilia A, respectively [3–7]. The application of AAV vectors to treat neurological diseases has recently attracted particular interest [8,9]; however, the delivery of exogenous genes by AAV vectors across the blood–brain barrier (BBB) and into the central nervous system (CNS) is a major challenge limiting the development of AAV-based therapeutics targeting the CNS system.

Several strategies for AAV capsid evolution have been developed to achieve higher efficiency in crossing the BBB. For example, a Cre recombination-based AAV targeted evolution (CREATE) method was applied to yield AAV9 variants with enhanced CNS transduction in mouse brain, e.g. AAV-PHP.B and AAV-PHP.eB [10]. These variants have an inserted peptide between amino acids Q588 and A589 in the variable region (VR) VIII of wild-type (wt) AAV9 VP3 [10,11]. Recently, a tropism redirection of AAV by cell-type-specific expression of RNA(TRACER) method for AAV evolution was developed based on the recovery of bulk capsid library RNA expressed in a cell-type-specific manner from nontransgenic animal tissue [12]. Among the generated AAV9 variants with higher efficiency for infecting the CNS, the variant AAV9P31, which includes an additional sequence (WPTSYDA) inserted between Q588 and A589 of the wt AAV9 VP3 VR-VIII, exhibited efficient CNS tropism with 385-fold higher EGFP expression in the mouse CNS and 1000-fold higher EGFP expression in the mouse spinal cord compared to wt AAV9 [12].

The specific receptor is a key determinant for AAV infection. Adeno-associated virus receptor (AAVR), a glycosylated protein containing five polycystic kidney disease (PKD) repeat domains (PKD1-5) [13], was first identified as a protein receptor for multiple AAV serotypes [14]. AAVR interacts with AAV1/2 by PKD2 contacting the spike of the AAV1/2 capsid, whereas AAVR binds with AAV5 by PKD1 lying on the plateau region of the AAV5 capsid [15,16]. Moreover, the GPI-linked protein LY6A was reported to play an essential role in AAV-PHP.B/PHP.eB crossing the BBB in selected mouse models [17–19]. A structural analysis revealed that one LY6A molecule binds at the icosahedral 3-fold axis of the AAV-PHP.B/PHP.eB particles, in which three VP3 capsomers clamp one LY6A [20]. Although the structural study revealed that the footprint of LY6A was associated with the inserted peptide in AAV-PHP.B/PHP.eB VP3 VR-VIII, icosahedral averaging within cryo-EM reconstruction resulted in only a block density at the icosahedral 3-fold axis instead of a traceable density for the polypeptide of LY6A [20].

A very recent work showed that the GPI-linked protein carbonic anhydrase IV (Car4) is an essential receptor for AAV9P31 and AAV9P36 crossing the BBB in a mouse model [21]. Car4 is localized on the luminal surface of brain endothelial cells throughout the cortex and cerebellum, and its sequence and structure are conserved in *Mus musculus* and *Homo sapiens* [22–25]. Biochemical results and structure prediction suggested that Car4 binds at the icosahedral 3-fold axis [21]. However, the molecular details of the AAV9P31-Car4 interaction remain unclear. In this paper, we report the atomic structures of AAV9P31 and the AAV9P31-Car4 complex by employing an improved block-based reconstruction method [26] to dissect the mechanism of the recognition of AAV9P31 by Car4. By combining mutagenesis studies, we have successfully identified key residues within the interfaces. This discovery serves as a foundation for further modifications of AAV9P31, potentially enabling its application in therapeutics for human neurological diseases.

## Results

### Structure of AAV9P31

**Fig 1:**
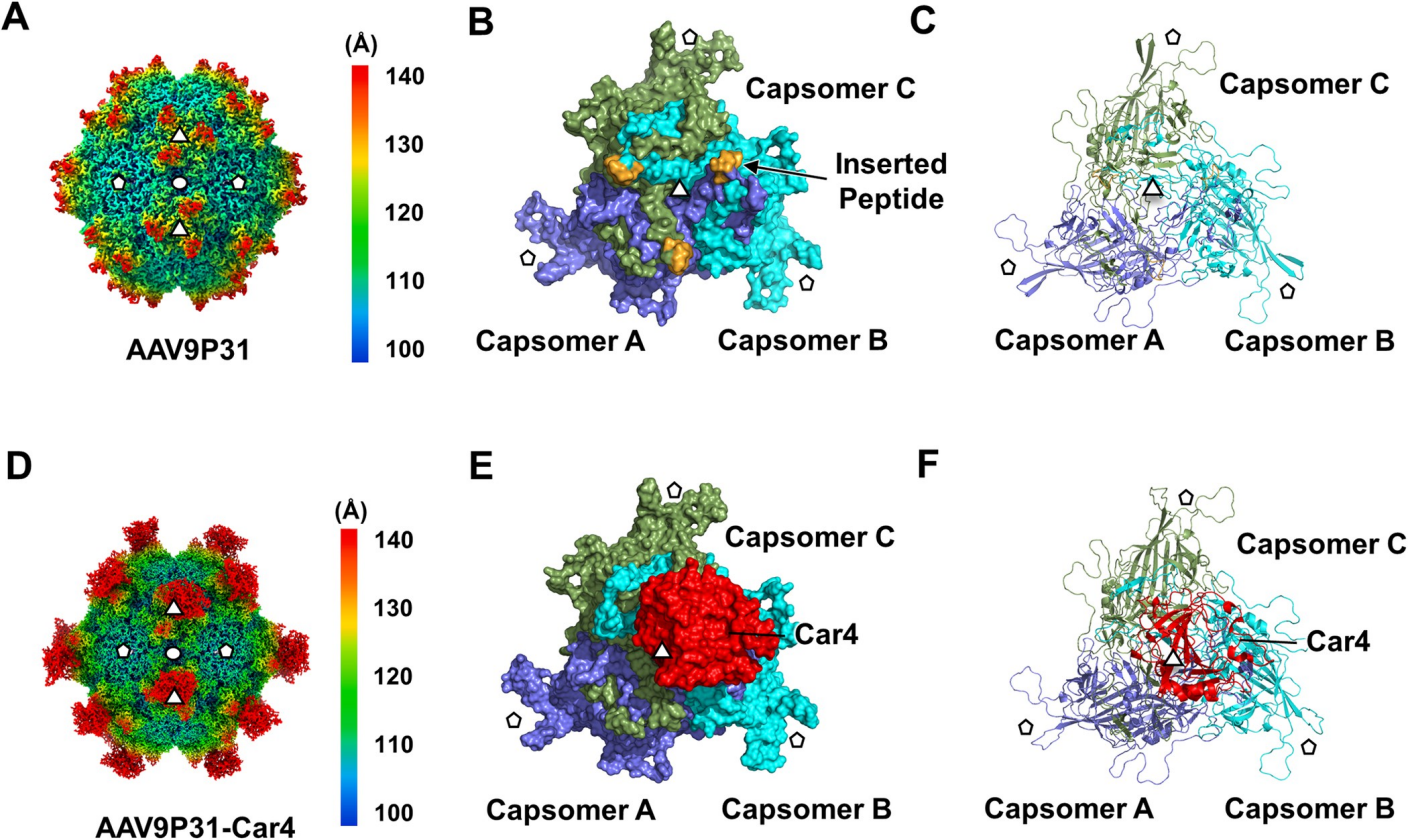
Cryo-EM reconstruction of AAV9P31 and AAV9P31-Car4. (**A**) The reconstructed cryo-EM map of AAV9P31. The radial distance color scheme is used to indicate the density (<100 Å: blue; 110–130 Å: from cyan to yellow; >140 Å: red). **(B)** The trimer of AAV9P31 VP3 in the threefold axis is colored by surface. **(C)** Cartoon representations of **(D)**. The inserted peptide of AAV9P31 is colored bright orange in **(B)** and **(C)**. **(D)** Overall cryo-EM map of the AAV9P31-Car4 complex. The radial distance color scheme is used to indicate the density by color (<100 Å: blue; 110–130 Å: from cyan to yellow; >140 Å: red). **(E)** The trimer of AAV9P31-Car4 in the threefold axis is colored by surface. **(F)** Cartoon representations of **(E)**. Capsomer A is colored slate blue; capsomer B is colored cyan; capsomer C is colored smudge green; Car4 is colored red **(B, C, E, F)**. The 5-, 3- and 2-fold icosahedral axes of symmetry are indicated with a pentagon, triangles, and an oval, respectively **(B, C, E, F)**.

The structure of AAV9P31 was determined by cryo-EM with a final resolution of 1.76 Å at a 0.143 cutoff of Fourier shell correlation (FSC) (S1A and S1B Fig, and S1 Table), and the side chains of VP3 capsomers were generally well defined (S2A–S2D Fig). The overall architecture of AAV9P31 and wt AAV9 (PDB: 7WJW) [20] share high similarities on most of the surface regions (with a root-mean-square deviation (RMSD) of 0.569 Å across all Cɑ atoms in VP3 residues), including a channel-like structure at the five-fold axes, a depression at the twofold axes and three protrusions surrounding each threefold axis (Fig 1A and 1B). The major difference is found in the VP3 VR-VIII region (S3A Fig). AAV9P31 has an additional seven inserted residues (WPTSYDA) between AAV9 VP3 Q588 and D589, which are numbered 588a to 588g (S3B Fig). The main chain of the inserted peptide W588a-A588g is well defined in the density map (S3C Fig).

### Block-based reconstruction (BBR) algorithm for reconstructing the AAV9P31-Car4 complex

**Fig 2:**
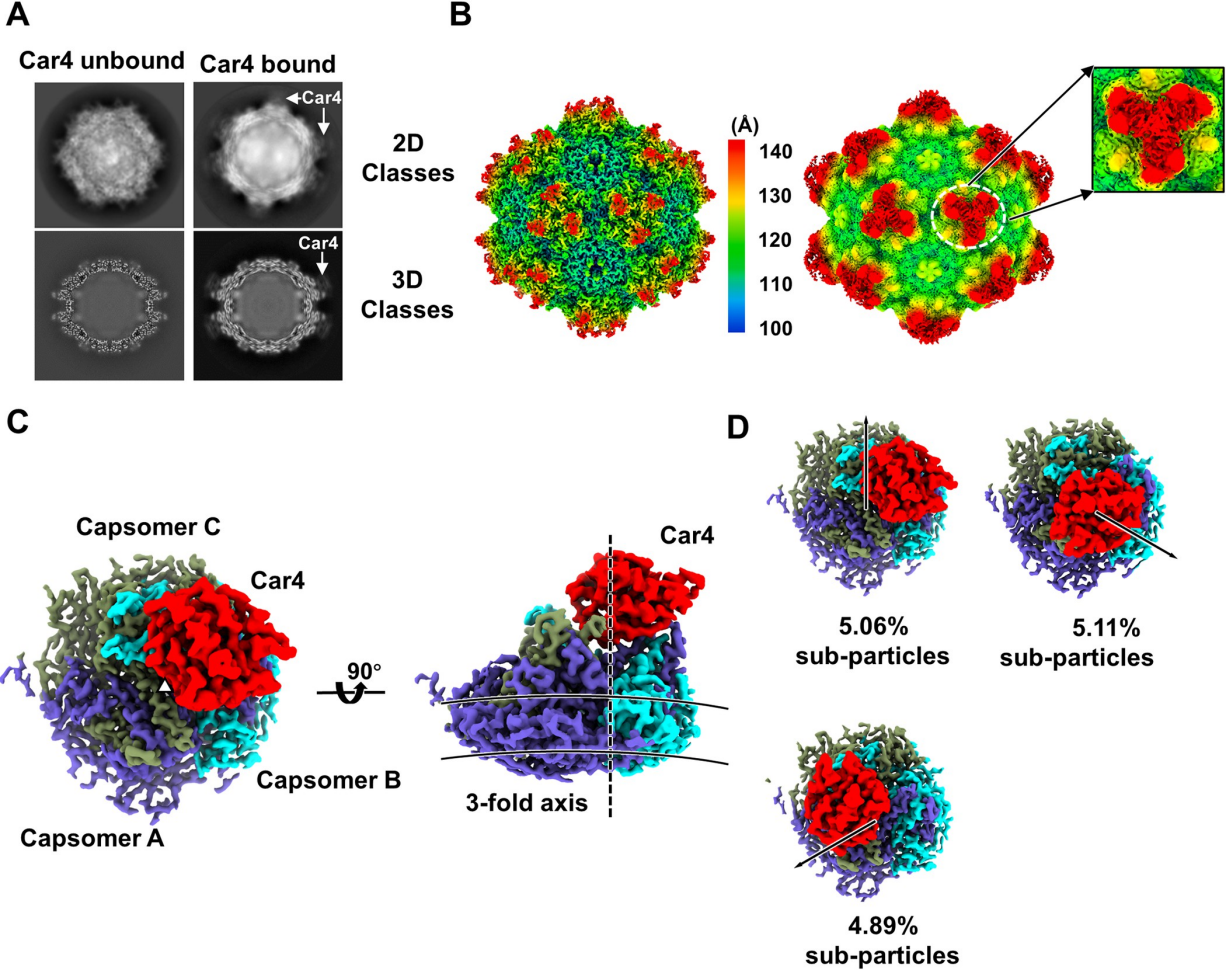
Block-based reconstruction of the 3-fold axis of AAV9P31-Car4. **(A)** 2D and 3D classification of the unbound and bound states of AAV9P31. The density of Car4 is indicated by a white arrowhead. The box size is 440 pixels, and the pixel size is 0.8433 Å. **(B)** Density map of icosahedral reconstructed AAV9P31-Car4 (unsharpened) shown by counter levels of 2.8σ (left) and 0.5σ (right). The magnified figure of the white circle shows the fuzzy electron density of Car4. The radial distance color scheme is used to indicate the density by color (<100 Å: blue; 110–130 Å: from cyan to yellow; >140 Å: red). **(C)**, **(D)** Density of three-fold axis blocks isolated from the AAV9P31-Car4 map. The icosahedral three-fold axis is indicated at its approximate position by the dotted line. The top view and side view are shown in **(C)**. The approximate inner and outer boundaries of the shell are marked by two solid arcs. Three classes of reconstructed blocks are shown in **(D)**. Capsomer A is colored slate blue; capsomer B is colored cyan; capsomer C is colored smudge green; and Car4 is colored red **(C, D)**.

To determine the binding details of Car4 on AAV9P31, AAV9P31 particles were incubated with purified Car4 (amino acids W22 to S277) for 5 minutes before cryo-EM sampling and data collection. In the 2D and 3D classification, additional density was found at the icosahedral three-fold axis compared to the native AAV9P31, indicating the binding of Car4 to AAV9P31 (Fig 2A). The initial cryo-EM single particle reconstruction was applied with icosahedral symmetry and yielded a final cryo-EM map with a resolution of 1.76 Å at an FSC cutoff of 0.143 (Figs 2B, S1C and S1D). Similar to a previous report of LY6A-AAV-PHP.B/PHP.eB study, additional densities were present at the icosahedral three-fold axis of the AAV9P31 capsid upon Car4 incubation (Figs 2B and S4). However, the current reconstruction strategy imposing icosahedral symmetry still failed to obtain the structure of Car4 at near-atomic resolution. In conventional algorithms for reconstructing an icosahedron, the three-fold axes are typically treated as three identical symmetric modules. Nevertheless, if a three-fold axis can only associate with an asymmetric receptor protein, the restoration of the three-fold axis deviates from the established symmetry. Consequently, achieving a three-dimensional reconstruction of the receptor density for the entire particle during icosahedron reconstruction becomes unattainable. Instead, computations must be conducted independently on each three-fold axis, treating them as sub-particles.

To overcome the aforementioned obstacle, we employed an improved block-based reconstruction (BBR) algorithm to calculate the receptor bound at the icosahedral axes (S5 Fig). The BBR method was developed by the group of Xinzheng Zhang [27]. In this method, large object can be split into smaller blocks by symmetry expansion so that sub-particles can be reconstructed and refined by single particle algorithms [27]. In total, 13,604,676 subparticles at the icosahedral three-fold axes were extracted by symmetry expansion of the particles (S5B Fig). After extracting all sub-particles from the micrographs, they were classified into sixteen classes. Five of sixteen classes showed clear signals of bound Car4, one of sixteen exhibited fuzzy noise at the position binding Car4, and the other 10 classes showed signals for VP3 alone (S5C Fig). The classes with Car4 signals are similar but have 120° rotations between them (Figs 2D and S5D–S5F). There are 2,067,911 particles in the 5 classes with the Car4 signal (15.2% of all extracted particles), indicating that each AAV9P31 virion binds only three Car4s on average. The final resolution of the three-fold axis block is 2.28 Å at 0.143 cutoff of FSC (S1E and S1F Fig).

### Overall structure of the AAV9P31-Car4 complex

**Fig 3:**
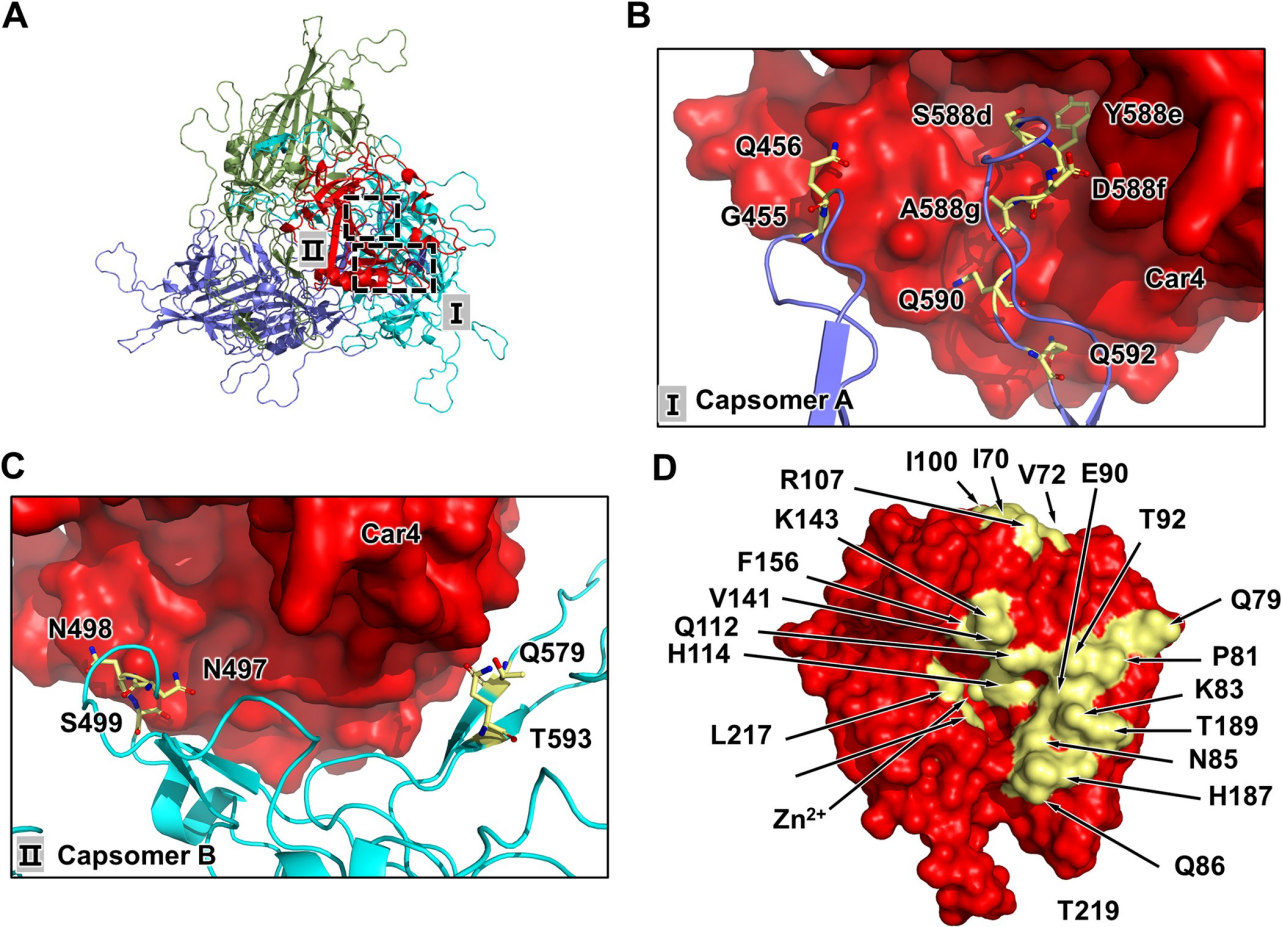
Interactions between AAV9P31 and Car4. **(A)** Cartoon representation of trimeric AAV9P31 capsomers in complex with Car4 from the top view. **(B)** and **(C)** Views of the binding interfaces of capsomer A and capsomer B, with residues that contact Car4 indicated. Car4 is shown on the surface; capsomers A, B or C are shown as cartoons; residues involved in the interfaces are shown in stick representation. **(D)** The residues in Car4 at the interfaces. The residues are indicated and shown in yellow. The letters on the Car4 surface are presented in bold white, and others are presented in bold black. AAV9P31 capsomers A and B are colored in slate blue and cyan, respectively. Bound Car4 is shown in red. Residues at the interfaces are shown in yellow.

In the structure of AAV9P31-Car4, Car4 asymmetrically interacts with the protrusion of three VP3 capsomers protruding at the icosahedral 3-fold axes (Figs 1D–1F and 2C). The binding pattern is totally different from that in our previously reported AAV9-AAVR complex (PDB: 7WJX; EMD-32551), which has three AAVRs binding at the virus three-fold axes [20]. For easy representation, we named the VP3 capsomers at the 8, 4 and 12 o’clock positions capsomers A, B and C, respectively (Fig 2C). The 3D classification in BBR shows that the bound Car4 has major contacts with two capsomers, either A/B, B/C or C/A, with very similar ratios (5.11%, 5.06% and 4.89%) and conserved binding behaviors in the three BBR groups (Figs 2D and S5D–S5F). Given that the BBR groups have 120-degree rotational symmetry about the 3-fold axis, we use the group in which Car4 interacts with capsomer A/B to represent the other two groups (Fig 1D–1F). Among the two interacting capsomers, capsomer A contributes the major interacting regions with the bound Car4 (Fig 3A–3C and S2 Table). The inserted peptide of capsomer A deeply inserted into the catalytic pocket of the bound Car4. Notably, the Car4 residues spanning L145_Car4_-D154_car4_, which were not identified in the previous work [23], can be clearly defined in the density (S6 Fig).

### Interactions of AAV9P31 with Car4

In AAV9P31-Car4 complex, the contact surface on Car4 is 1926.2 Å^2^ compared to its entire 23,459 Å^2^ accessible surface, indicating a strong virus-receptor interaction. The residues of Car4, including I70_Car4_, V72_Car4_, Q79_Car4_, P81_Car4_, K83_Car4_, N85_Car4_, Q86_Car4_, E90_Car4_, T92_Car4_, G94_Car4_, Q112_Car4_, V141_Car4_, F156_Car4_, H187_Car4_, T189_Car4_, L217_Car4_ and T219_Car4_, were found to be involved in the interaction with AAV9P31, as analyzed by the contact program in the CCP4 suite (distance cutoff of 4.0 Å) [28] (Fig 3D and S2 Table). The interfaces are well defined in the density (S7 Fig)

**Fig 4:**
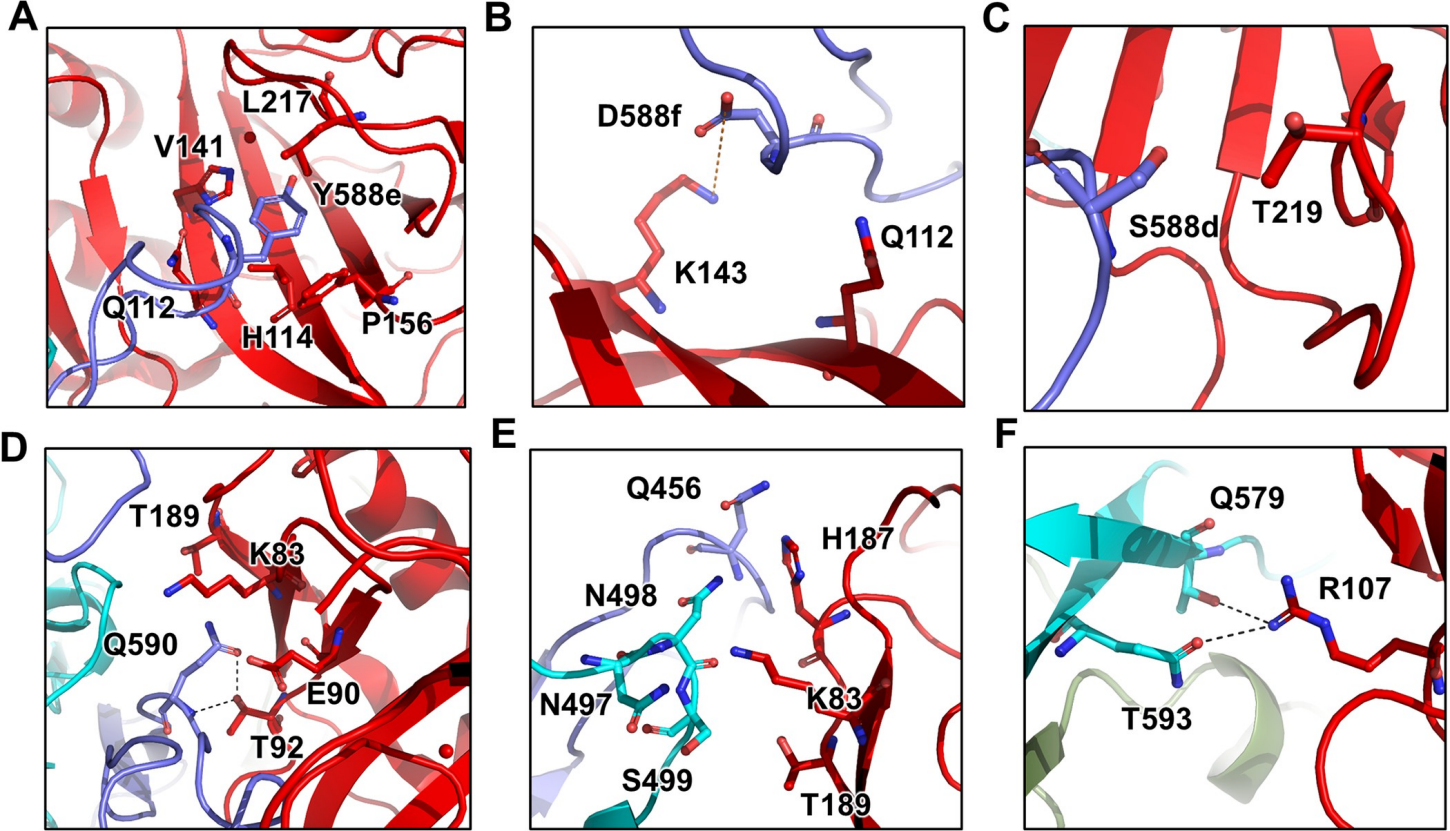
Detailed contacts between AAV9P31 VP3 and the bound Car4. The residues of **(A)** Y588e; **(B)** D588f; **(C)** S588d; **(D)** Q590; **(E)** Q456, N497, N498 and S499; and **(F)** Q579 and T593 that interact with Car4 are shown. Capsomer A is colored slate blue; capsomer B is colored cyan; car4 is colored red. The salt bridge is indicated by the orange dotted line; the hydrogen bonds are shown by the black dotted lines.

The interacting residues of AAV9P31 are located on the protrusions and ridges of capsomers A/B near the icosahedral three-fold axes. The contacting residues of capsomer A are mainly distributed in VR-VIII. The residue Y588e_AAV9P31-A_ of capsomer A has close contacts with the residues Q112_Car4_/H114_Car4_/V141_Car4_/P156_Car4_/L217_Car4_ of the bound Car4 (Fig 4A). Residue D588f_AAV9P31-A_ of capsomer A form salt bridges with K143_Car4_ in the positively charged region of the Car4 active center (Fig 4B). Moreover, residue S588d_AAV9P31-A_ is involved in the interaction with T219_Car4_ (Fig 4C). Q590_AAV9P31-A_ interacts with K83_Car4_, E90_Car4_, T92_Car4_ and T189_Car4_ (Fig 4D). Moreover, Q456_AAV9P31-A_, N497_AAV9P31-B_ and N498_AAV9P31-B_ of capsomer B approaches K83_Car4_, H187_Car4_ and T189_Car4_ of the bound Car4 to stabilize the virus-receptor interaction (Fig 4E), as well as Q579_AAV9P31-B_ and T593_AAV9P31-B_ form hydrogen bonds with R107_Car4_ (Fig 4F).

Structural comparison revealed that binding with Car4 induces conformational changes in the inserted peptides in AAV9P31 capsomers A and B (S8 Fig). Binding with Car4 induces conformational change of the inserted peptide (region I) and the loop adjacent to it (region II) in AAV9P31 capsomer A (S8A Fig). In region I, the backbone of the inserted peptide in the Car4-bound state is pulled toward Car4 from its native state by ~2.7 Å (S8B Fig). The side chains become ordered in their three-fold block reconstruction. In region II, the backbone of the loop from T451_AAV9P31-A_-T460_AAV9P31-A_ in the Car4-bound AAV9P31 is pushed from its original position into the capsid by ~1.8 Å (S8C Fig). In contrast, the contacts with AAV9P31 capsomers do not result in obvious shifts of the bound Car4. The bound state structure of Car4 and the individual Car4 structure (PDB: 2ZNC) share an RMSD of 1.1543 Å across all Cα atoms. A conformational change of Car4 can be observed in the loop region formed by residues G94_Car4_-C98_Car4_ (S6C Fig). However, because this loop region is distant from the interacting surface, the conformational change is unlikely to be related to virus-receptor interactions.

### Mutagenesis study

In order to examine the impacts of the interacting residues on AAV9P31-Car4 recognition, we systematically performed alanine scanning for Car4 interface residues to assess the binding affinities with AAV9P31, employing the surface plasmon resonance (SPR) method. Subsequently, we also conducted mutations on the residues situated at the interaction interfaces of the AAV9P31 capsid surface, aiming to elucidate their influence on AAV infection.

**Fig 5:**
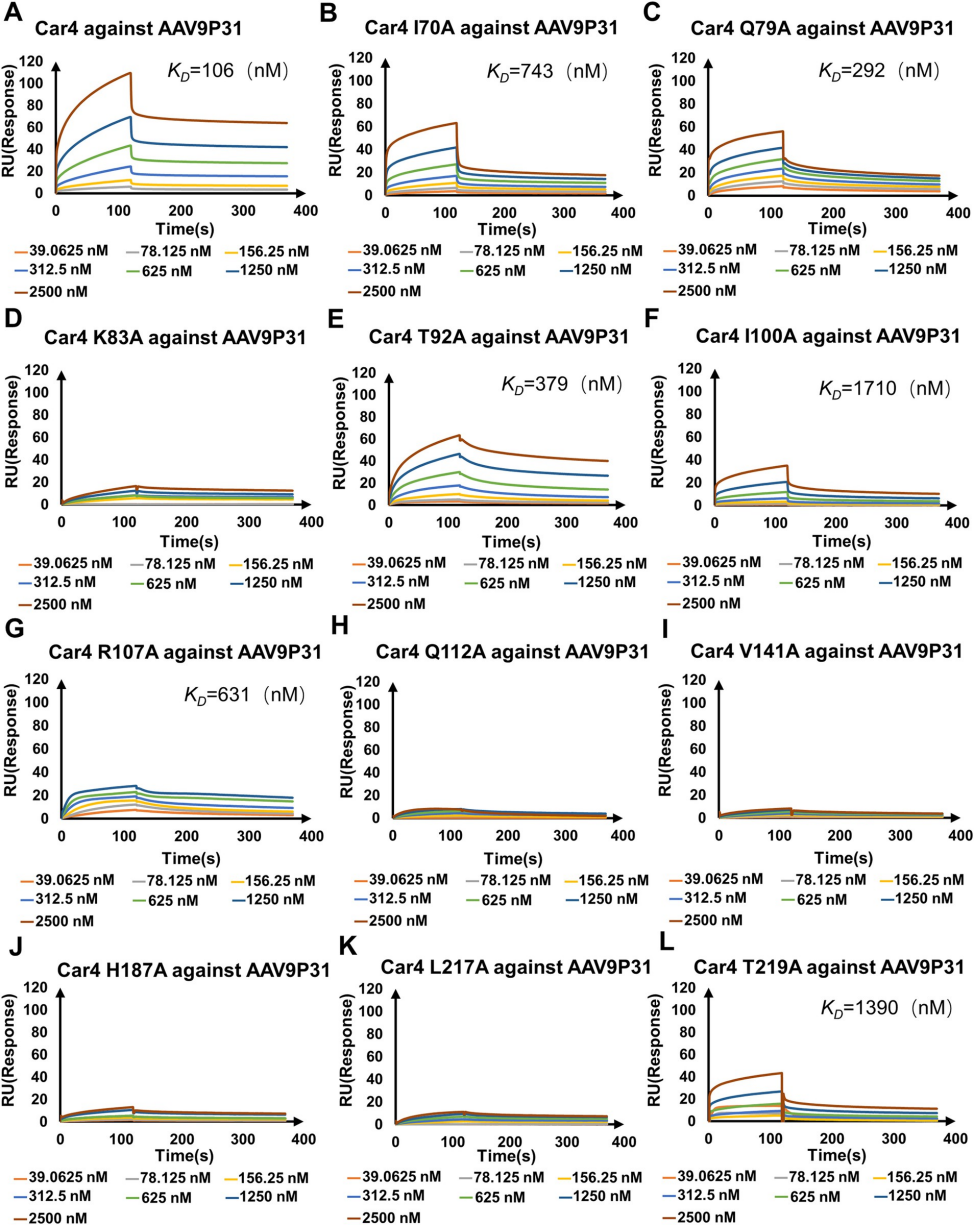
Mutagenesis study of Car4 for AAV9P31 binding assays. **(A)-(L)** Wild-type Car4 and 11 mutants were tested for binding capability to the AAV9P31 capsid by BIAcore 8K sensorgrams. The concentrations of the analytes are indicated with different colors in each panel. RU, resonance units.

The residues of Car4 involved in the mutagenesis study included I70A_Car4_, Q79A_Car4_, K83A_Car4_, T92A_Car4_, I100A_Car4_, R107A_Car4_, Q112A_Car4_, V141A_Car4_, H187A_Car4_, L217A_Car4_ and T219A_Car4_ (Figs 5B–5L and S9). Among them, the F156A_Car4_ mutant could not be expressed, possibly because F156 is close to the catalytic center of Car4 and plays an essential role in the correct folding of Car4.

The results show that wt Car4 binds to AAV9P31 with an equilibrium dissociation constant (K_D_) value of 106 nM (Fig 5A and S3 Table), and cannot interact with AAV9 (S11 Fig). All mutants of Car4 showed attenuated binding affinities with AAV9P31. Among them, K83_Car4_, I100_Car4_, R107_Car4_ and H187_Car4_ generated decreased RU values in SPR (Fig 5D, 5F, 5G and 5J), which is consistent with their interactions with AAV9P31 engaged by their large side chains. The replacements of Q112_Car4_ and L217_Car4_ as alanine residues decreased the interaction with the hydrophobic side chain of Y588e_AAV9P31_, resulting in almost nondetectable response curves in SPR (Fig 5H and 5K). It is likely that the T219_Car4_ and S588d_AAV9P31_ interactions were also disrupted by the mutation (Fig 5L). Moreover, the mutants I70A_Car4_, Q79A_Car4_ and T92A_Car4_ modestly attenuated the binding affinity with AAV9P31 (K_D_ values were 743nM, 292 nM and 379 nM, respectively) (Fig 5B, 5C and 5E, and S3 Table).

We also substituted the AAV9P31 capsid residues on the interface with alanine residues and tested the transduction activities of the mutated viruses. The results revealed a notable over 50% reduction in infection for mutants Q579A_AAV9P31_, S588dA_AAV9P31_, Q590A_AAV9P31_, and T593A_AAV9P31_ compared to the wild-type AAV9P31. Additionally, mutants N497A-N498A_AAV9P31_, D588fA_AAV9P31_, and Y588eA_AAV9P31_ demonstrated a complete loss of cell infection capability (S10 Fig). These results are consistent with structural observations that these seven AAV9P31 capsid residues form extensive intermolecular contacts with T92, R107, K143, P156, H187, T189, L217 and T219 in Car4 to stabilize the AAV9P31-Car4 interaction.

### Interactions of AAV9P31 with human carbonic anhydrase IV

**Fig 6:**
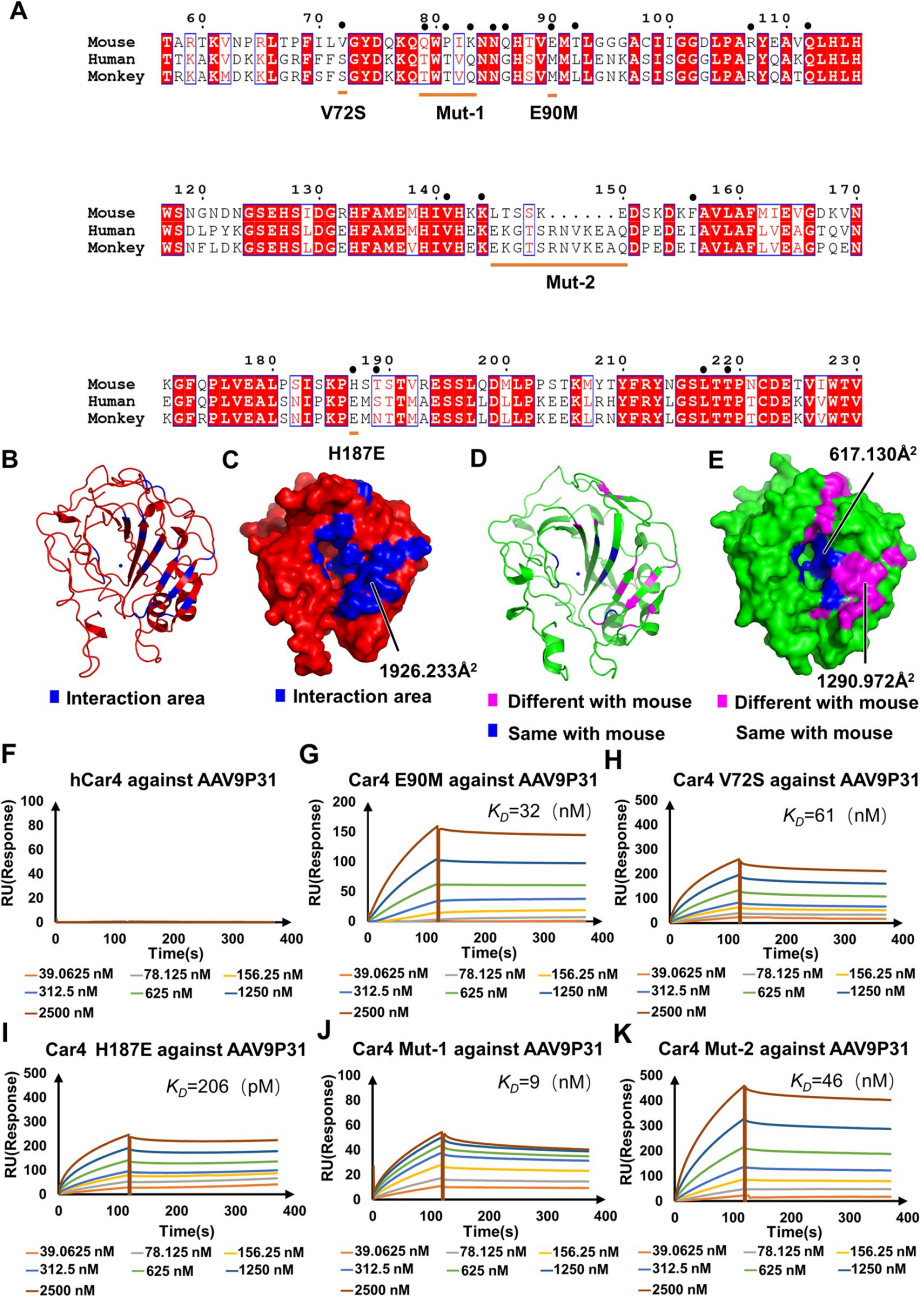
Car4 comparisons among *Mus musculus*, *Homo sapiens* and *Rhesus macaque*. **(A)** Sequence comparison among Car4 ofmice (T57 to V221), humans (T58 to V237) and monkeys (T61 to V240). The mutagenesis sites are shown by orange lines; the interacting residues on Car4 are indicated by large black dots. The red high light marks the most conserved region; yellow lines indicate the site mutations from mouse to human. The structures of Car4 (red, PDB: 2ZNC) and hCar4 (green, PDB: 1ZNC) are shown in cartoon **(B), (D)** and surface **(C), (E)** representation. The specific residues where AAV9P31 interacts with Car4 are colored blue while the different residues on hCar4 are colored magenta. Binding assays of wild-type hCar4 and Car4 mutants (V72S, E90M, H187E, Mut-1, Mut-2) with AAV9P31 in BIAcore 8K sensorgrams are shown in **(F)-(K)**. Concentrations are presented in different colors. The K_D_ values are available in S3 Table.

In mammals, carbonic anhydrases (CAs) belong to a single gene family known as α-Cas, which consists of 16 different isoforms, 13 of which (CA I, II, III, IV, VA, VB, VI, VII, IX, XII, XIII, XIV, and XV) are enzymatically active [29]. Among them, CA IV (Car4) is a GPI-linked membrane bound protein initially purified from bovine lung [30] and also found in the brain [31], kidney [32], heart [33], eye [34], and erythrocytes [35] that is responsible for the hydration of CO_2_. Because Car4 molecules are conserved in their structures and sequences throughout vertebrates (including mice, nonhuman primates and humans [23,36]) (Fig 6A–6E), we speculated that human Car4 (hCar4) may also bind AAV9P31 similar to *Mus musculus* Car4. However, the SPR results showed that wt hCar4 did not show detectable binding with AAV9P31 (Fig 6F), indicating that the potential application of AAV9P31 in humans might need further optimization, particularly in its interface with Car4.

Sequence alignments of *Car4* in *Mus musculus*, *Homo sapiens* and *rhesus macaque* indicated several key nonconserved residues that are involved in or close to the AAV9P31-Car4 interface (Fig 6A). We next mutated these residues in Car4 to their counterparts in hCar4 and measured their binding affinities with AAV9P31 by SPR. These mutants include three single residue mutations, V72S_Car4_, E90M_Car4_ and H187E_Car4_, as well as two short peptide mutations, Mut-1_Car4_ and Mut-2_Car4_ (Fig 6A). In sharp contrast to the nondetectable binding of wt hCar4 with AAV9P31, all five mutants retained binding (Fig 6G–6K). The mutant V72S_Car4_ exhibited binding to AAV9P31 with a K_D_ value of 60.7 nM, which is even higher than the binding of wt Car4 with AAV9P31 (Fig 6G and S3 Table). Because V72_Car4_ has a hydrophobic side chain, changing V72_Car4_ to a serine would increase its interaction with the hydrophilic side chain of Q456_AAV9P31-B_. Moreover, E90M_Car4_ resulted in a highly improved binding affinity with K_D_ value of 32.3 nM, possibly by introducing more hydrogen bonds with Q590_AAV9P31_ (Fig 6H and S3 Table). The greatest increase in binding to AAV9P31 was observed with the mutant H187E_Car4_, which resulted in an ~10^3^ fold increase in binding affinity with a K_D_ value of 206 pM (Fig 6I and S3 Table). We hypothesize that the mutation of H187_Car4_ to a glutamate residue may provide improved interactions with G455_AAV9P31-B_, Q456_AAV9P31-B_ and N498_AAV9P31-B_. Furthermore, two peptide mutants, Mut-1_Car4_ and Mut-2_Car4,_ also increased the binding affinity with AAV9P31 (K_D_ = 45.9 nM and 8.7 nM, respectively) (Fig 6J, 6K and S3 Table). The inserted peptide of Mut-2_Car4_ is near the protrusions surrounding the 3-fold axis of AAV9P31, which might form more interactions with the capsid.

Meanwhile, non-conserved residues engaged in Car4 binding underwent mutations in hCar4 to align with their counterparts in Car4. This includes S72V_hCar4_, M90E_hCar4_, E187H_hCar4_, and Mut-1_hCar4_. Notably, none of the four mutants exhibited interactions with AAV9P31, as evidenced by SPR experiments (S12 Fig). The reason why AAV9P31 cannot bind to hCar4 may be more complicated than the influence of these counterpart residues.

## Discussion

Recognition of the specific receptor is a key determinant for AAV infection and understanding the mechanism of the AAV-receptor provides valuable information for further optimization of AAV that could be used in gene therapies. In a set of previous works, the cryo-EM structures of AAVR in complex with AAV1 [16], AAV2 [15,37,38], AAV5 [16,39], AAV9 [20], AAV-PHP.eB [20] and AAVGo.1 [40] were studied. In addition to recognition by AAVR, AAVs require more specific receptors (or entry factors) for infection within the CNS, particularly for passing through the BBB. In recent years, LY6A [18], Car4 and LY6C [36] were found to be essential for allowing recombinant AAV to pass through the BBB in mouse models. In this study, we determined the structures of AAV9P31 alone or in complex with Car4. Consistent with the functional experiments, the residues Q112_Car4_, V141_Car4_, H187_Car4_, and L217_Car4_ along with N498_AAV9P31-B_ and Y588e_AAV9P31-A_ at the interface are essential for virus-receptor binding, forming hydrogen bonds and salt bridges. The inserted peptide in the AAV9P31 VR-VIII loop is very important for receptor binding. As a result, the interfaces offer a wide choice of residues to mutate to potentially increase the efficiency of BBB crossing.

The current design of AAV capsids targeting the CNS depends on several screening strategies, M-CREATE [10] and TRACER [12], and mainly focuses on the Q588-A589 insertion in AAV9 VP3. Such strategies, similar to the successful improvement of AAV9 in mice for CNS targeting, remain uncertain of success in nonhuman primates [41,42]. We also demonstrated that although Car4 in humans and mice is conserved in sequence and structure, AAV9P31 cannot biochemically bind hCar4 *in vitro*, which further indicates that the novel engineered AAV capsid variants generated from current directed evolution strategies are still largely limited in terms of host species. However, several hCar4-derived mutations on Car4 presented improved binding affinity against AAV9P31. In particular, an H-to-E mutation at position 187 in Car4 resulted in a 10^3^-fold increase in binding. Because H187_Car4_ interacts with N498_AAV9P31-B_, G455_AAV9P31-B_ and Q456_AAV9P31-B_ on AAV9P31 VP3 capsomers, it is conceivable that the modifications on these three VP3 residues to provide improved contacts with H187_Car4_ allow the AAV9P31 variant to be recognized by hCar4. From a structural standpoint, valuable information is obtained by superimposing hCar4 onto Car4 in the AAV9P31 bound state. It is observed that Q590_AAV9P31_ approaches a non-conserved region of hCar4, potentially leading to clashes with T58_hCar4_, Q60_hCar4_, and L69_hCar4_ (S13 Fig). Consequently, Q590_AAV9P31_ represents a key point for AAV modification that can be employed in primates or humans.

Unlike the clearly elucidated of AAV-AAVR interaction, the binding between novel cellular receptors and engineered AAVs with 7-mer insertions occurs asymmetrically at 3-fold axes on the capsids [20,21]. This is a major obstacle in reconstructing the binding receptors located at the icosahedral axes of virus particles. A set of cryo-EM reconstruction methodologies have been investigated to overcome this obstacle, including focused classification to determine the structure of transferrin receptor bound with canine parvovirus [43] and symmetry-relaxation to analyze the interaction of coxsackievirus B3 (CVB3) with coxsackie and adenovirus receptor (CAR) inserted in nanodiscs [44]. In this study, we extended the application of the block-based reconstruction (BBR) [26,45] method, which was originally developed to improve the local resolution for large-scale particles, to study the asymmetric virus-receptor binding mode. The success of the structural study of the AAV9P31-Car4 complex by the BBR method presents an important solution for asymmetric receptor binding on icosahedral viruses.

In summary, the atomic structure of the AAV9P31-Car4 complex expands our knowledge of the molecular mechanism of AAV BBBcrossing. The precise virus–receptor interactions revealed in this study may facilitate the design and optimization of recombined AAV9 vectors by pinpointing specific engineerable candidate residues to produce particles with both higher efficiency for BBB crossing and the ability to cross species from mouse to human.

## Materials and methods

### Virus production and purification

Triple-plasmid transfection using polyethyleneimine reagent (PEIMAX) (No. 24765, Polysciences, USA) was used to produce recombinant AAV9P31 according to a previously reported protocol [16]. The pAAV9P31-GFP, pRepCap encoding AAV9P31 or mutants virus protein, and pHelper plasmids were cotransfected into HEK293T cells. For the large-scale production of AAVs, cells were seeded in 150 mm dishes at a density of 1 × 10^7^ cells/dish for 24 hr prior to transfection and transfected with 12 μg of pHelper plasmid, 8 μg of AAV9P31 pRepCap plasmids, and 5 μg of pAAV9P31-GFP plasmids at 70% confluency. At 72 hr. post-transfection, cells were harvested by centrifugation at 500 × g at 4°C for 5 min. The pellet was collected and resuspended in lysis buffer containing 50 mM Tris-HCl, pH 8.0, 150 mM NaCl. The suspension was subjected to three freeze-thaw cycles in dry ice/ethanol and a 37°C water bath. Then, benzonase (100 units/ml) and 0.5% sodium deoxycholate were added, and the cell suspension was incubated for 1 hour at 37°C. Following centrifugation at 5,000 × g for 20 min at 4°C, the supernatant containing the AAV9P31 crude lysate was collected. For AAV9P31 infection assay, AAV crude lysate genome copy titers were determined by real-time quantitative PCR (qPCR) using primers specific for the GFP gene sequences. The primers used were as follows: qpcr-GFP-F, TCTTCAAGTCCGCCATGCC; qpcr-GFP-R, TGTCGCCCTCGAACTTCAC.

The crude lysate was diluted with 10 mM Tris-HCl (pH 8.0) buffer to a final volume of 10 ml and then bottom loaded on a discontinuous gradient of 15%, 25%, 40% and 60% iodixanol in a 39 ml ultracentrifuge tube (Quick-Seal 342414, Beckman, USA). After ultracentrifugation at 350,000 × g at 18°C for 1 hr, 3 ml fractions in the 40% lower layer and 0.5 ml of the 60% upper layer were collected. The viral titers in each fraction were determined by negative staining. The fractions with the highest titers were desalted using a 100 kDa cutoff ultrafiltration tube (15 ml; Millipore, USA), and the buffer was changed to PBS. Purified AAV9P31 was stored at -80°C until use.

### Purification of Car4

DNA encoding the truncated Car4 (22–277) (NCBI Gene ID: 12351) with an N-terminal GST-tag in a pGEX-6p-1 vector was transformed into *Escherichia coli* BL21 (DE3) cells and cultured in Luria-Bertani (LB) medium containing 100 μg/ml ampicillin at 37°C. When the OD_600_ of the culture reached 0.6, protein expression was induced by the addition of both isopropyl β-D-thiogalactoside (IPTG) and zinc sulphate (ZnSO_4_) at a final concentration of 0.5 mM, followed by another 18 hr. of cell culture. The cells were harvested by centrifugation at 4,000 rpm for 15 min. The pellet was resuspended in lysis buffer (20 mM HEPES, pH 8.0; 150 mM NaCl) and then homogenized by sonication. The fusion protein was then isolated from its crude lysate by GST affinity chromatography (Qiagen, Holland) and digested overnight by HRV 3C Protease at 16°C without shaking in lysis buffer. The eluted protein was further purified by size exclusion using Superdex 75 (GE Healthcare, USA). Mutated Car4 constructs were generated using the Fast Mutagenesis System (Transgene, China), and the mutated proteins were purified as described above. The purified proteins were concentrated to 2 mg/ml for storage at -80°C until use.

### Sample preparation and cryo-EM data collection

A 5 μl aliquot of AAV9P31 was loaded onto a glow-discharged, carbon-coated copper grid (Cu 1.2/1.3+C, 300 mesh; Quantifoil) bearing an ultrathin layer of carbon and incubated for two minutes. The liquid was then sucked away by a filter and 3 μl of Car4 was loaded on the same grid, and incubated for another five minutes with 100% humidity. The grid was then blotted for 4.5 s with a blot force of 0 in 100% relative humidity and plunge-frozen in liquid ethane using a Vitrobot Mark IV (FEI, USA). Cryo-EM data were collected with a 300 kV Titan Krios electron microscope (FEI, USA) and a K3 direct electron detector (FEI, USA). A series of micrographs were collected as movies and recorded with -2.2 to -0.5 μm defocus at a calibrated magnification of 105,000×, resulting in a pixel size of 0.8433 Å per pixel. Statistics for data collection and refinement are summarized in S1 Table.

### Image processing and three-dimensional icosahedral reconstruction

After data collection, each movie was motion corrected and dose-weighted micrographs were abtained by MotionCor2 [46]. The contrast transfer function parameters were estimated by CTFFIND4 [47]. Then all micrographs with CTF fitting resolution > 4 Å were excluded and 6,068 good images were obtained for subsequent data processing. Particles were auto picked and extracted in Relion-3.1.2 [48,49]. After multiple rounds of 2D classification and 3D classification with C1 symmetry, 226,724 good particles were selected and subjected to 3D autorefinement with I1 symmetry yielding a reconstruction with a resolution of 2.13 Å. To push a higher resolution, we further used global and local CTF refinement to refine the beam tilt and per-particle CTF and brought the resolution to 1.89 Å. The final reconstruction used Ewald sphere correction with negative curvature yielding a reconstruction with a resolution of 1.76 Å. AAV9P31 particles without Car4 were reconstructed and refined in CryoSPARC-4.0.3 [50]. Following the same steps as above, the final reconstruction ended in 1.76 Å resolution.

### Sub-particles classification and reconstruction in the 3-axis region

Block-based reconstruction was used to extract blocks near the 3-fold axes and icosahedral symmetry was applied [26]. The coordinates of a sub-particle were first determined in USCF Chimera. After employing symmetry expansion and dropping blocks located outside the micrographs, 13,604,676 blocks were selected and re-extracted with a smaller box size (160 pixels). Blocks were reconstructed with the “relion_reconstruct” command to generate a reference map and the reference was low pass filtered to 60 Å for one round of 3D classification which applied C1 symmetry without alignment. Sixteen classes were generated, six of which included notable receptor density. Those classes contained receptors that are similar in structure but the rotational difference between them is approximately 120°. Blocks with the same receptor binding site were combined and subjected to 3D autorefinement with C1 symmetry, yielding reconstruction of Car4-VP3 complexes with resolutions of 2.59 Å, 2.59 Å and 2.28 Å, respectively.

### Model building and refinement

All maps were built in Coot and refined in Phenix real space refine [51,52]. The AAV9 cryo-EM structure (PDB:7WJW) and Car4 crystal structure (PDB:2ZNC) were used as references for AAV9P31 and AAV9P31-Car4 model building. All figures were prepared in Chimera, ChimeraX and PyMOL [53–55].

### Surface plasmon resonance (SPR)

SPR analyses were carried out using a BIAcore 8K (GE Healthcare, USA) with a flow rate of 30 μl/min at 25°C in PBST buffer. AAV9P31 particles suspended in sodium acetate buffer (pH 4.0) were immobilized on a CM5 sensor chip by amide coupling. Different concentrations of the recombinant WT or mutated Car4 proteins flowed over the chip and between each sample we used glycine-HCl (10mM glycine, pH 2.0) for chip surface regeneration. The binding affinity was determined, and curves were generated by BIAEvaluation software (GE Healthcare, USA).

### AAV infection assays

Recombinant AAV vectors carrying a GFP expression cassette were used to evaluate viral transduction efficiency. Twenty-four hours prior to infection, HEK293T cells were seeded at 80% confluency in a 10cm plate and maintained in Dulbecco’s modified Eagle’s medium (DMEM) supplemented with 10% FBS and penicillin-streptomycin (100 U ml^−1^) at 37°C in 5% CO_2_. DNA encoding the full length Car4 (NCBI Gene ID: 12351) with a C-terminal 3 × Flag-tag was inserted in a pBMCL vector, which was a gift from Prof. Lei Chen in Peking University. The full length Car4 was transiently expressed in HEK293T cells by transfecting with 16 μg of plasmid DNA. Twelve hours later, Car4-expressing cells were transferred to 96-well plates with 2 × 10^4^ per well and maintained in DMEM supplemented with 10% FBS, penicillin-streptomycin (100 U ml^−1^) at 37°C in 5% CO_2_. Cells were infected with AAV9P31 or mutants at an MOI of 1 × 10^6^ viral genome (vg) per cell in triplicate. At 48 h post-infection, GFP expression levels were measured by flow cytometry using a BD LSRFortessa analyzer (BD Biosciences, USA).

## Supporting information

S1 Fig Resolution of AAV9P31 and AAV9P31-Car4. Fourier shell correlation (FSC) of the final 3D reconstruction following gold standard refinement using RELION [48,49]. The resolutions corresponding to an FSC of 0.143 are shown for **(A)** unbound AAV9P31, **(C)** AAV9P31-Car4 complex and **(E)** 3-fold block of AAV9P31-Car4. **(B), (D), (F)** The surface of the maps is colored from red to blue indicating the different local resolutions.
(PDF)

S2 Fig The key loop density of reconstructed AAV9P31. The unbound AAV9P31 **(A)** VR-I, **(B)** VR-III, **(C)** VR-IV and **(D)** VR-V fragment stick models fitted into electron density. VR-1: counter level = 1.0σ, carve = 1.5; VR-III: counter level = 2.0σ, carve = 1.5; VR-IV: counter level = 1.0σ, carve = 1.5; VR-V: counter level = 2.0σ, carve = 1.5. Electron density is presented as sweat mesh.
(PDF)

S3 Fig Structure and sequence alignment of AAV9 and AAV9P31. **(A)** The cartoon models of AAV9(gray) and AAV9P31(cyan) are aligned together. The red box shows the structural difference in variable region VIII between AAV9 and AAV9P31. The 5-, 3- and 2-fold icosahedral axes of symmetry are indicated with a pentagon, triangles, and an oval, respectively. **(B)** Sequence alignment of AAV9 and AAV9P31 in variable region VIII. The inserted peptide residues are highlighted in bright yellow. **(C)** The stick model of the inserted peptide (pink) in variable region VIII in AAV9P31 electron density.
(PDF)

S4 Fig Cryo-EM data-processing pipeline for the AAV9P31-Car4 complex. Overview of the cryo-EM data processing pipeline in RELION [48,49]. **(A)** Cryo-EM sample data collected in a 300 kV Titan Krios electron microscope with 0.8433 Å per pixel. **(B)** The micrographs are selected with the CTF estimated maximum resolution (threshold: <4.0 Å). **(C)** Particles are automatically picked from micrographs. **(D)** The particles selected by multiple rounds of 2D classifications. **(E)** Multiple rounds of 3D classifications with C1 symmetry, after which two classes were selected for the 3D refinement. **(F)** The AAV9P31-Car4 complex was reconstructed with I1 symmetry after local CTF refinement. The selected classes are colored gray and unselected classes are colored white.
(PDF)

S5 Fig Cryo-EM data-processing pipeline for AAV9P31-Car4 3-fold blocks. Overview of the cryo-EM data processing pipeline of block-based reconstruction in RELION [48,49]. **(A)** The I1 reconstructed 3D map of the AAV9P31-Car4 complex. **(B)** Blocks are isolated from the icosahedral 3-fold axes by symmetry expansion. **(C)** 3D classifications of 3-fold blocks. **(D)-(F)** Three different classes are refined in C1 symmetry. Car4 electron density presented as blue; noise presented as red.
(PDF)

S6 Fig Structural comparison between bound and unbound Car4. **(A)** The overall structures of the bound and unbound (PDB:2ZNC) states of Car4. **(B)** The newly solved loop region is enlarged, and shown in stick representarion in our electronic density. The red ring shows that the bound state Car4 swings considerably compared with the unbound state. The unbound state Car4 is colored light blue and the bound state is colored orange. The mesh is colored gray. **(C)** The magnified figure of the red circle in **(A)**. Loops of bound and unbound state Car4 are shown in stick representation.
(PDF)

S7 Fig Density of key residues at AAV9P31-Car4 interfaces. Left side of the panel shows the key interacting region in bound state AAV9P31 **(A)** 586–591, **(B)** 455–459, **(C)** 495–501 and **(D)** 590–592 fragments’ stick models fitted into electron density. The right side of the panel shows the key interacting region in Car4 **(E)** 79–86, **(F)** 111–115, **(G)** 186–190 and **(H)** 154–156 fragment stick models fitted into electron density. Electron density is presented as gray mesh. Capsomer A is shown in blue-slate; capsomer B is shown in cyan; Car4 is shown in red.
(PDF)

S8 Fig Conformational changes in the AAV9P31 capsid following Car4 attachment. **(A)** The structural change in the AAV9P31 capsid between the Car4-bound (red) and unbound AAV9P31 (gray) states, shown in cartoon representation. The approximate inner and outer boundaries of the shell are marked by two solid arcs. The icosahedral three-fold axis is indicated at its approximate position by the dotted line. Car4 is shown in red. **(B)** and **(C)** The two major conformational changes in regions I and II boxed in **(A)** are enlarged in **(B)** and **(C)**, respectively. Residues with conformational changes in Car4 and the AAV9P31 capsid are shown in stick representation with the same colors as in the cartoon diagrams.
(PDF)

S9 Fig All the yield proteins were tested by SDS–PAGE. **(A)** The peak in gel filtration chromatography by Superdex 75 (GE). **(B)-(F)** SDS–PAGE of mutants of Car4, hCar4, wild-type Car4 and hCar4. The molecular weights are labeled on each panel. Car4 or hCar4 is approximately 35 kDa.
(PDF)

S10 Fig AAV9P31 viral particles bearing capsid mutations at the receptor-binding sites impacted viral infection. HEK293T transduced by full length Car4 were infected with mutant AAV9P31 at an MOI of 1 × 10^6^ vg cell^−1^. GFP expression was determined 48 h post-transduction by flow cytometry. The percentages of cells infected by AAV9P31 mutants are plotted as means ± standard errors (n = 3).
(PDF)

S11 Fig The binding affinity between Car4 and AAV9. Wild-type Car4 was tested for binding capability to the AAV9 by BIAcore 8K sensorgrams. The concentrations of the analytes are indicated with different colors in each panel. RU, resonance units.
(PDF)

S12 Fig AAV9P31 and hCar4 mutants binding assay. **(A)-(D)** Human Car4 mutants were tested for binding capability to the AAV9p31 capsid by BIAcore 8K sensorgrams. The concentrations of the analytes are indicated with different colors in each panel. RU, resonance units.
(PDF)

S13 Fig Superposition of hCar4 and Car4 in the AAV9P31 bound state. **(A)** hCar4 is superposed onto Car4 in the AAV9P31-Car4 complex. hCar4 is colored wheat; Car4 is colored grey; AAV9P31 virus proteins are colored pink. **(B)** Magnification of the red box in the left panel to highlight the details of the residues. The distances between the residues are marked. The residues of Car4 involved in the interactions are colored limon; the counterpart residues in hCar4 are colored blue; Q590_AAV9P31_ is colored in red. All these residues are shown as sticks.
(PDF)

S1 Table Cryo-EM data collection, refinement and validation statistics. (PDF)

S2 Table Interactions between AAV9P31 and Car4. (PDF)

S3 Table Surface plasma resonance (SPR) experiment. (PDF)

S1 Data Excel spreadsheet containing, in separate sheets, the underlying numerical data and statistical analysis for figure panels Figs 5A, 5B, 5C, 5D, 5E, 5F, 5G, 5H, 5I, 5J, 5K, 5L, 6F, 6G, 6H, 6I, 6J, 6K, S10, S11, S12A, S12B, S12C and S12D. (XLSX)
