# Supplementary figures and images for: Structural basis of the recognition of adeno-associated virus by the neurological system-related receptor carbonic anhydrase IV
Source: PLoS Pathog. 2024 Feb 5;20(2):e1011953. doi: 10.1371/journal.ppat.1011953 (PMC10868842; doi:10.1371/journal.ppat.1011953)

**A**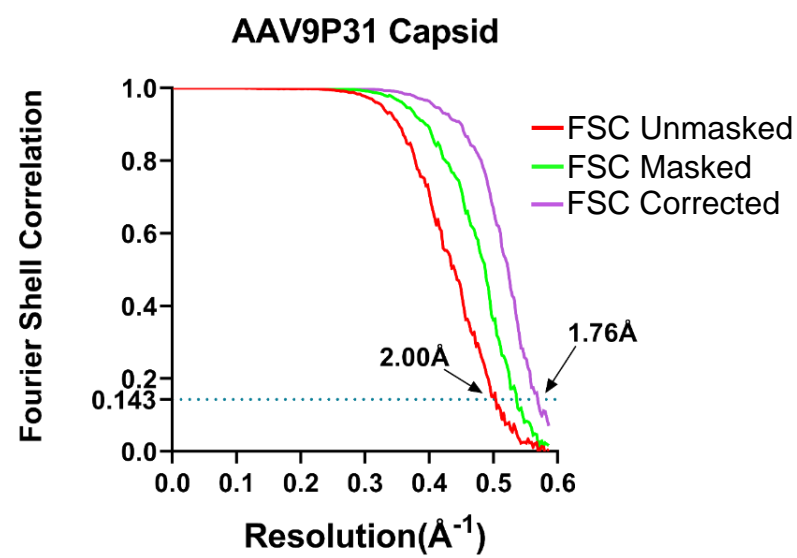**B**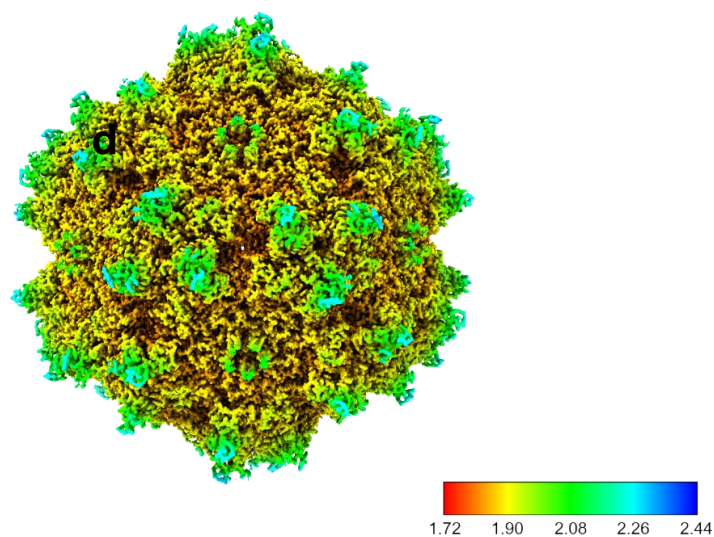**C**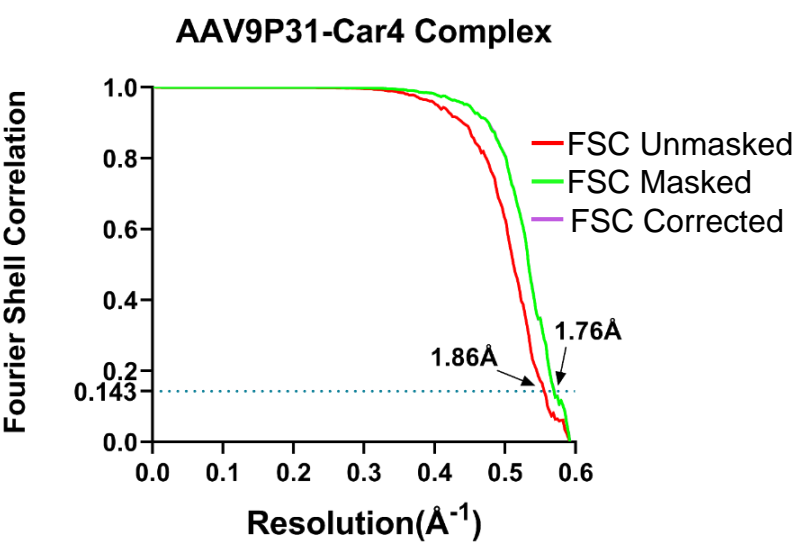**D**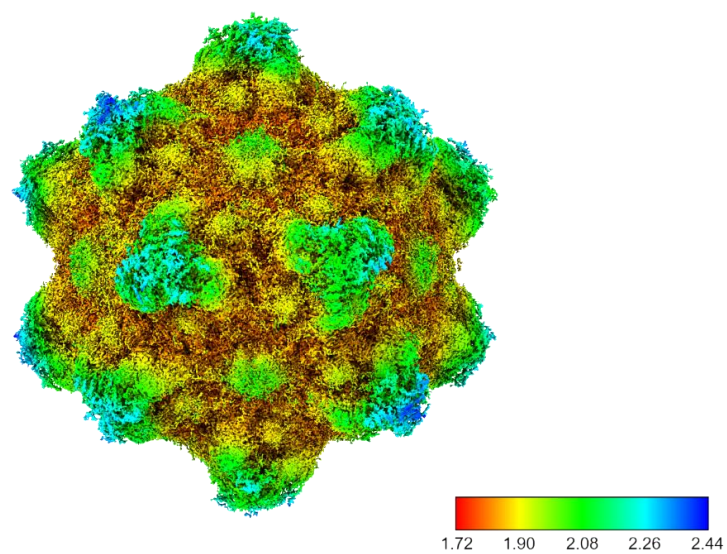**E**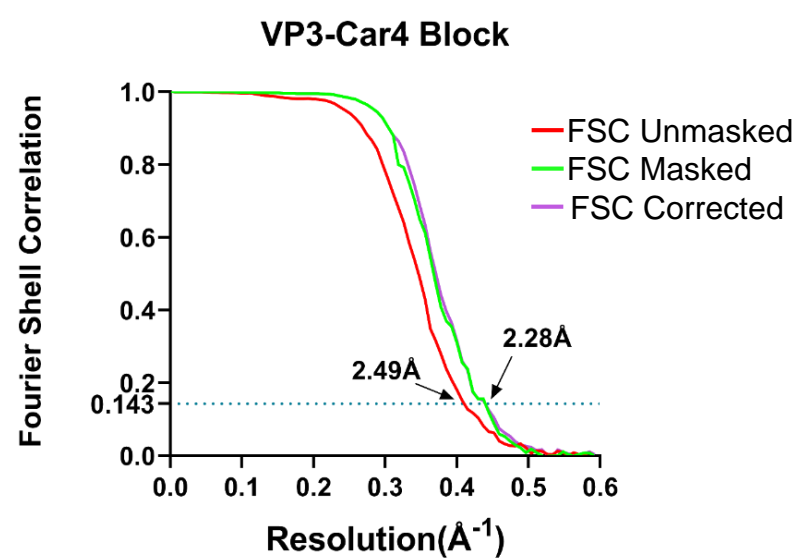**F**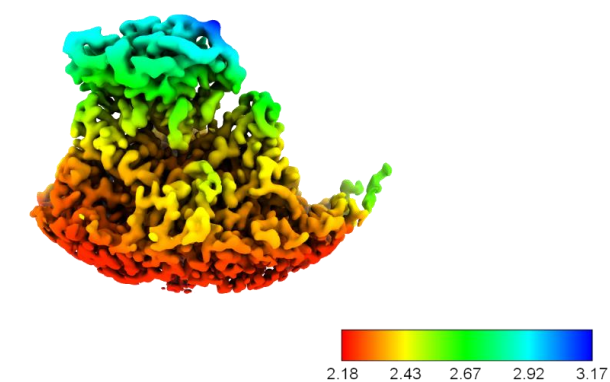

Supplement: S1 Fig — Fourier shell correlation (FSC) of the final 3D reconstruction following gold standard refinement using RELION [48,49]. The resolutions corresponding to an FSC of 0.143 are shown for (A) unbound AAV9P31, (C) AAV9P31-Car4 complex and (E) 3-fold block of AAV9P31-Car4. (B), (D), (F) The surface of the maps is colored from red to blue indicating the different local resolutions. (PDF) [file ppat.1011953.s001.pdf]

**A**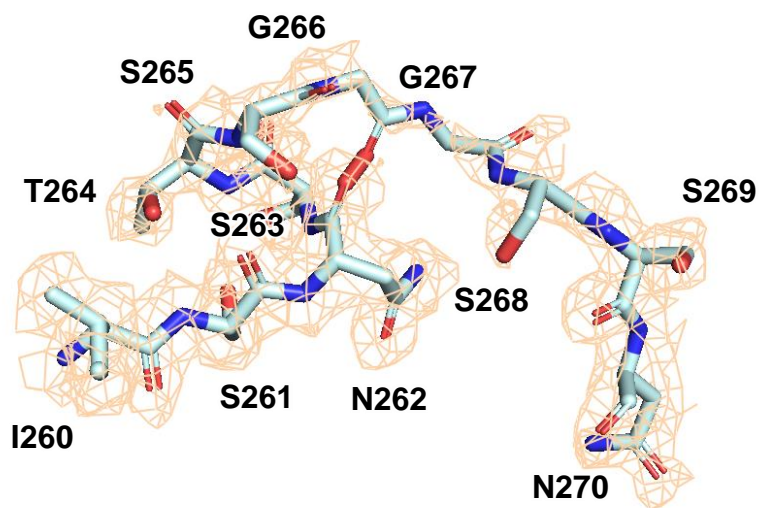**B**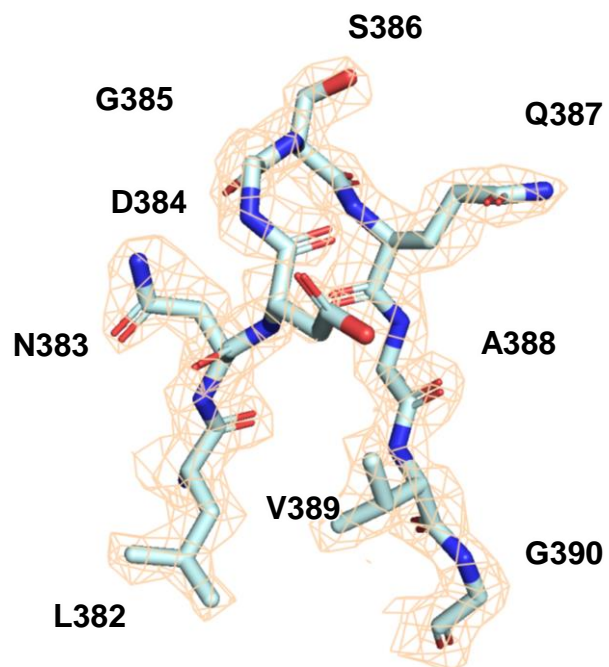**C**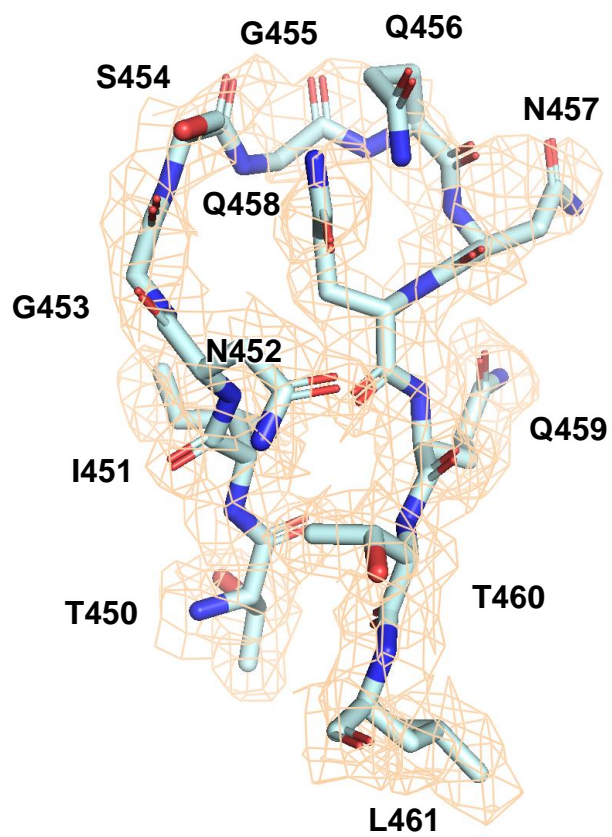**D**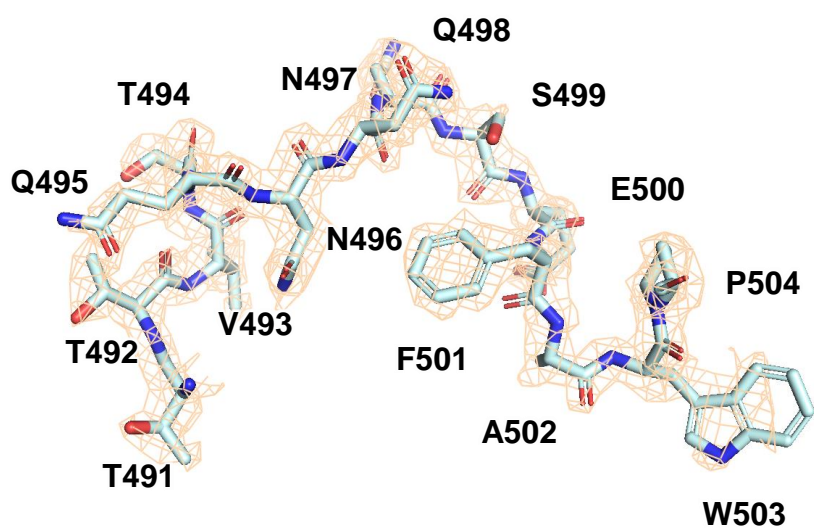

Supplement: S2 Fig — The unbound AAV9P31 (A) VR-I, (B) VR-III, (C) VR-IV and (D) VR-V fragment stick models fitted into electron density. VR-1: counter level = 1.0σ, carve = 1.5; VR-III: counter level = 2.0σ, carve = 1.5; VR-IV: counter level = 1.0σ, carve = 1.5; VR-V: counter level = 2.0σ, carve = 1.5. Electron density is presented as sweat mesh. (PDF) [file ppat.1011953.s002.pdf]

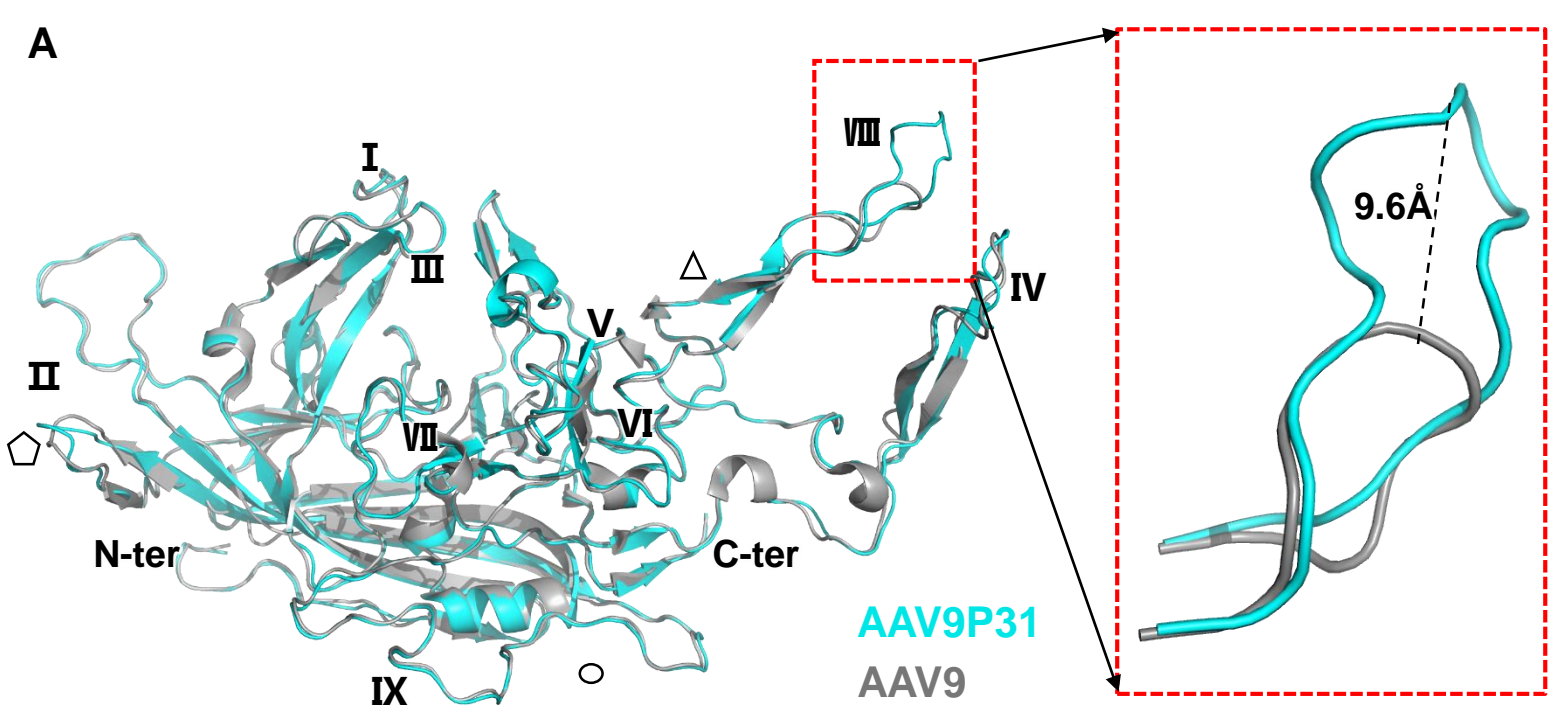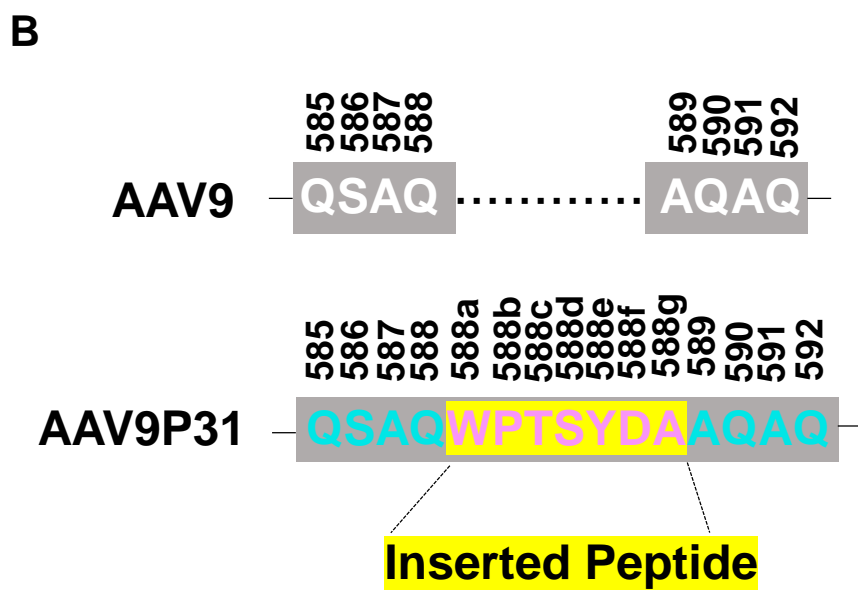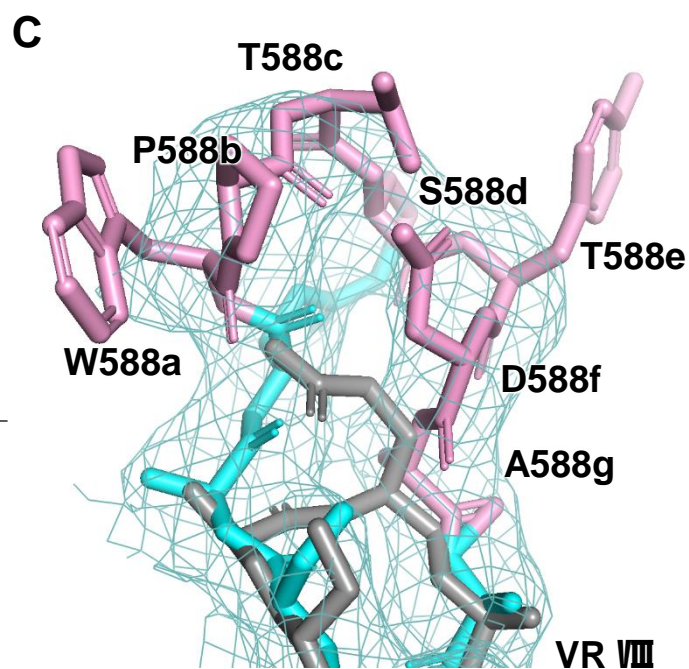

Supplement: S3 Fig — (A) The cartoon models of AAV9(gray) and AAV9P31(cyan) are aligned together. The red box shows the structural difference in variable region VIII between AAV9 and AAV9P31. The 5-, 3- and 2-fold icosahedral axes of symmetry are indicated with a pentagon, triangles, and an oval, respectively. (B) Sequence alignment of AAV9 and AAV9P31 in variable region VIII. The inserted peptide residues are highlighted in bright yellow. (C) The stick model of the inserted peptide (pink) in variable region VIII in AAV9P31 electron density. (PDF) [file ppat.1011953.s003.pdf]

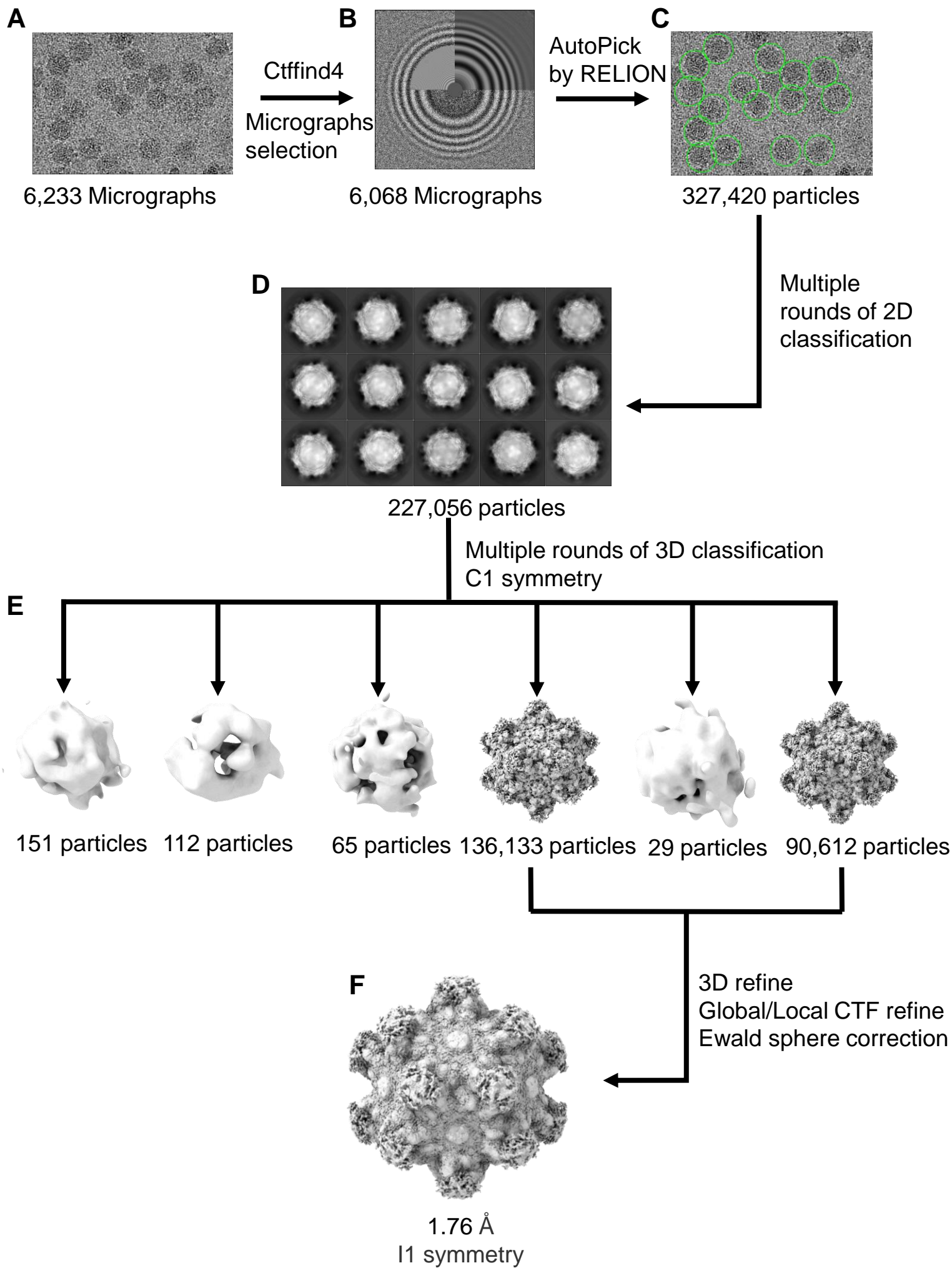

Supplement: S4 Fig — Overview of the cryo-EM data processing pipeline in RELION [48,49]. (A) Cryo-EM sample data collected in a 300 kV Titan Krios electron microscope with 0.8433 Å per pixel. (B) The micrographs are selected with the CTF estimated maximum resolution (threshold: <4.0 Å). (C) Particles are automatically picked from micrographs. (D) The particles selected by multiple rounds of 2D classifications. (E) Multiple rounds of 3D classifications with C1 symmetry, after which two classes were selected for the 3D refinement. (F) The AAV9P31-Car4 complex was reconstructed with I1 symmetry after local CTF refinement. The selected classes are colored gray and unselected classes are colored white. (PDF) [file ppat.1011953.s004.pdf]

**A**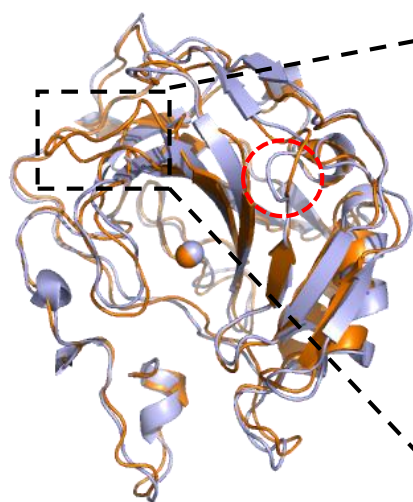

— Bound state  
— Unbound state

**B**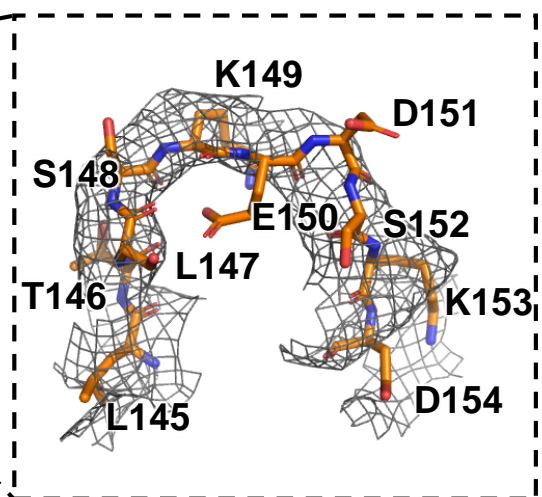**C**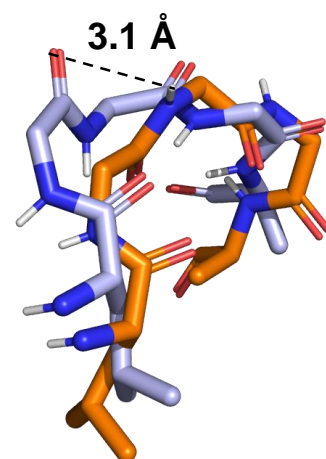

93-97aa:LGGA

Supplement: S6 Fig — (A) The overall structures of the bound and unbound (PDB:2ZNC) states of Car4. (B) The newly solved loop region is enlarged, and shown in stick representarion in our electronic density. The red ring shows that the bound state Car4 swings considerably compared with the unbound state. The unbound state Car4 is colored light blue and the bound state is colored orange. The mesh is colored gray. (C) The magnified figure of the red circle in (A). Loops of bound and unbound state Car4 are shown in stick representation. (PDF) [file ppat.1011953.s006.pdf]

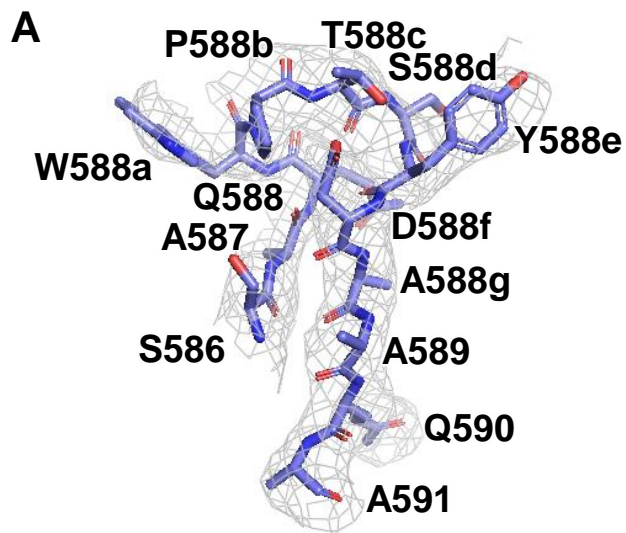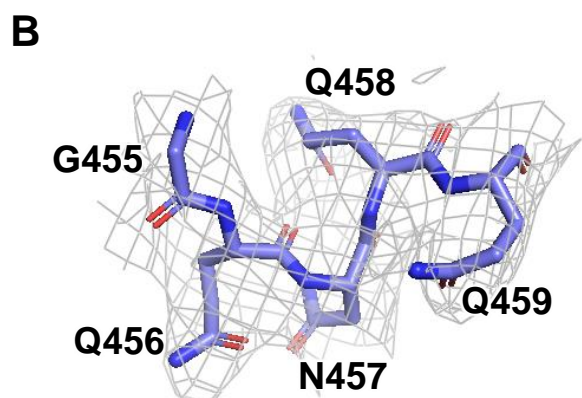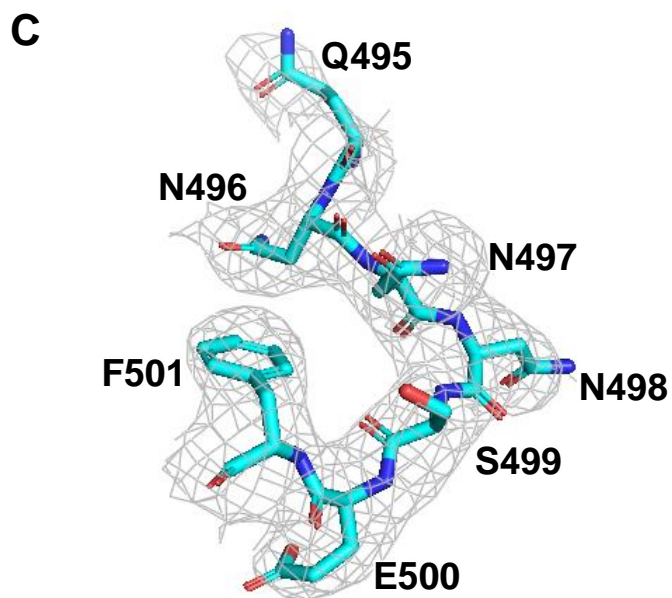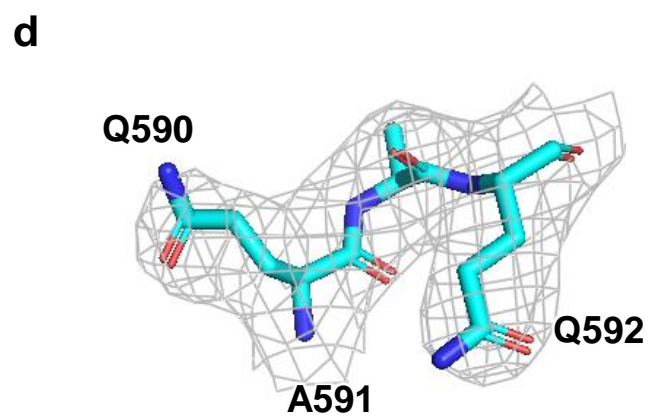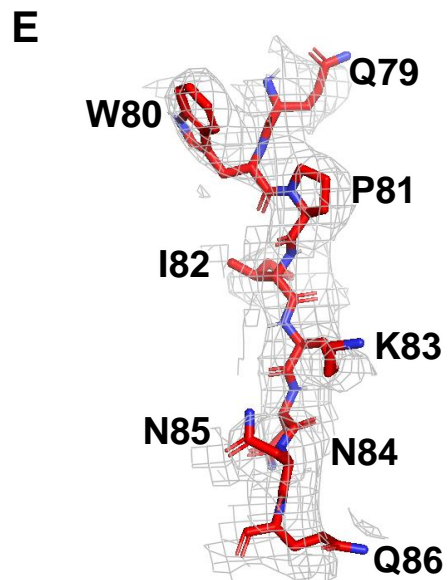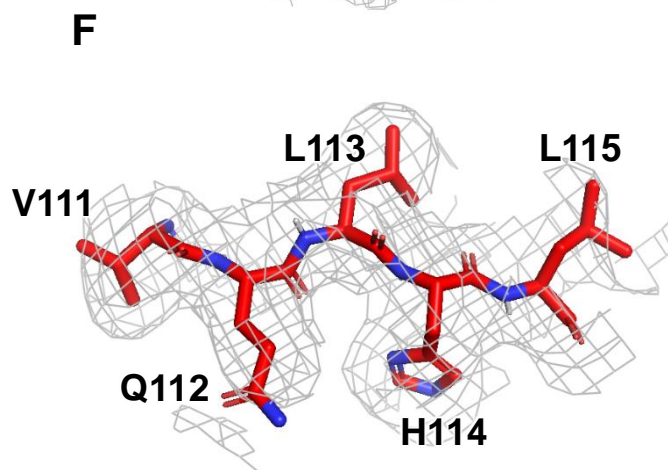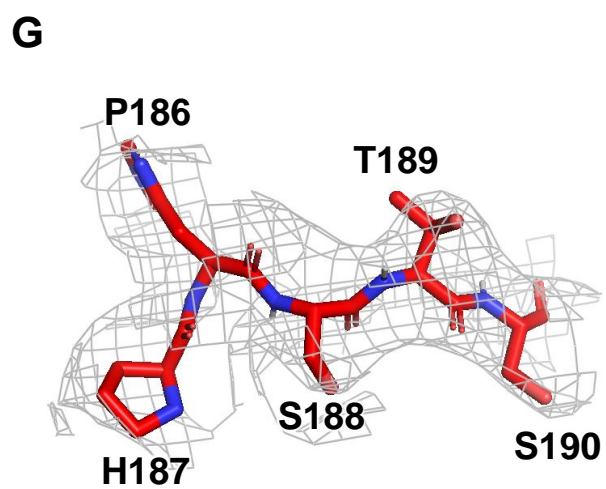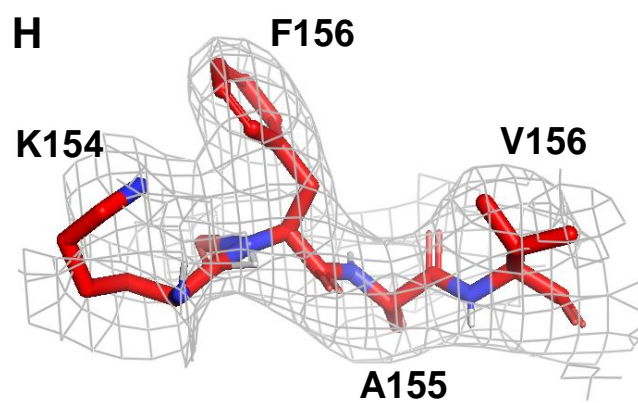

Supplement: S7 Fig — Left side of the panel shows the key interacting region in bound state AAV9P31 (A) 586–591, (B) 455–459, (C) 495–501 and (D) 590–592 fragments’ stick models fitted into electron density. The right side of the panel shows the key interacting region in Car4 (E) 79–86, (F) 111–115, (G) 186–190 and (H) 154–156 fragment stick models fitted into electron density. Electron density is presented as gray mesh. Capsomer A is shown in blue-slate; capsomer B is shown in cyan; Car4 is shown in red. (PDF) [file ppat.1011953.s007.pdf]

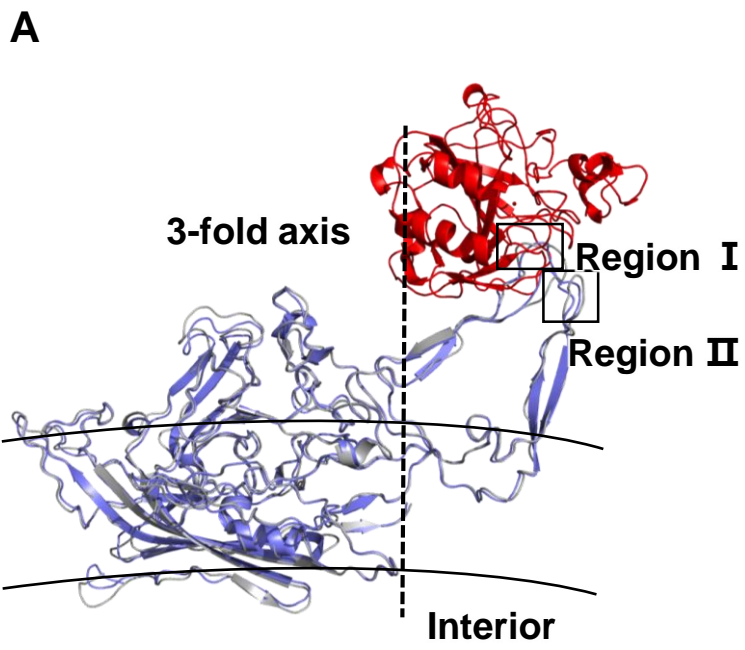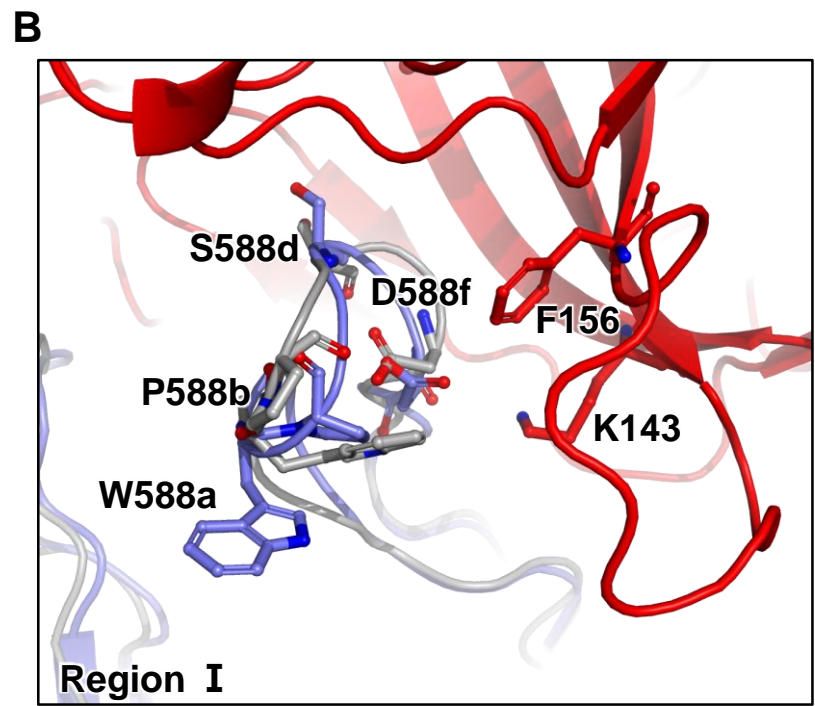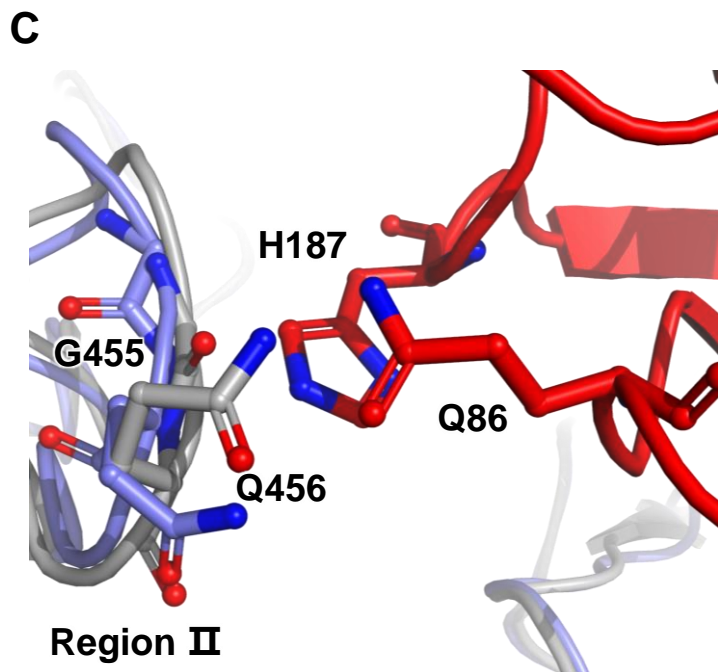

Supplement: S8 Fig — (A) The structural change in the AAV9P31 capsid between the Car4-bound (red) and unbound AAV9P31 (gray) states, shown in cartoon representation. The approximate inner and outer boundaries of the shell are marked by two solid arcs. The icosahedral three-fold axis is indicated at its approximate position by the dotted line. Car4 is shown in red. (B) and (C) The two major conformational changes in regions I and II boxed in (A) are enlarged in (B) and (C), respectively. Residues with conformational changes in Car4 and the AAV9P31 capsid are shown in stick representation with the same colors as in the cartoon diagrams. (PDF) [file ppat.1011953.s008.pdf]

**A**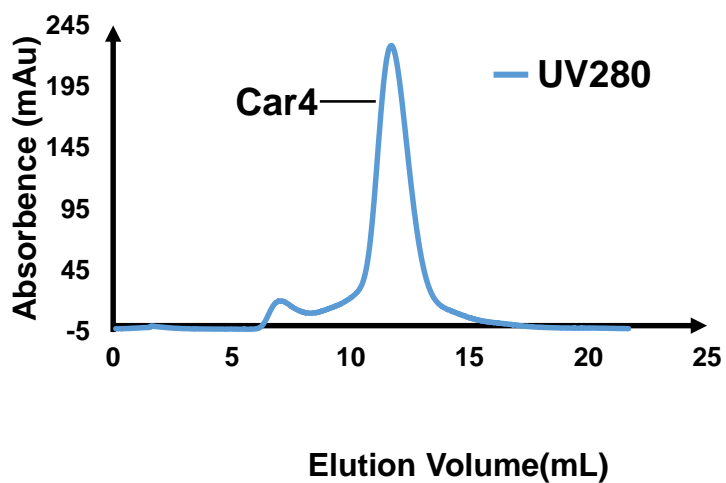**B**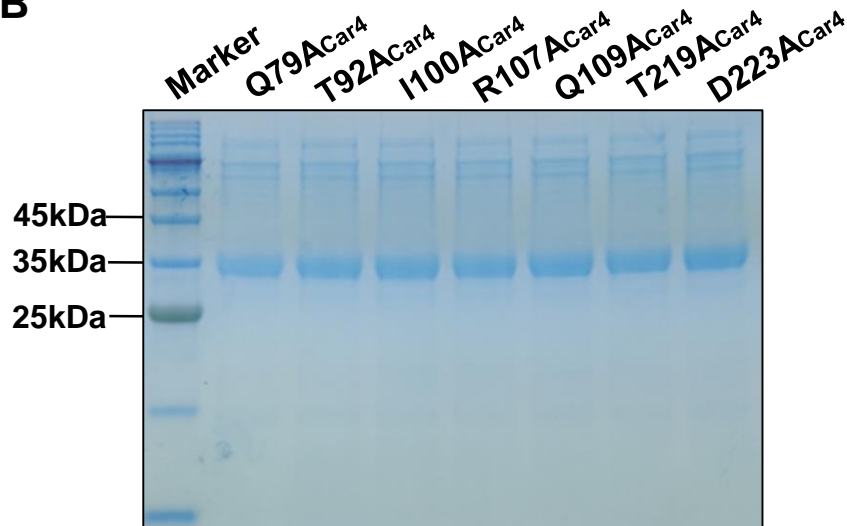**C**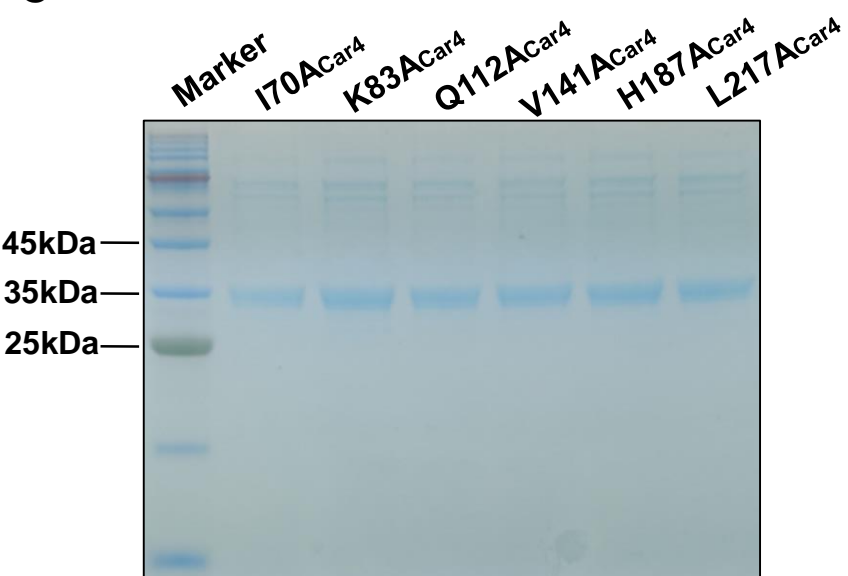**D**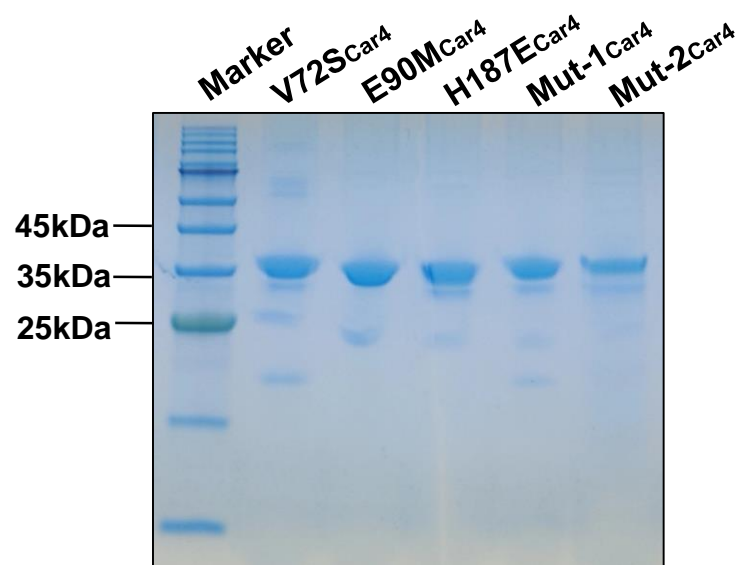**E**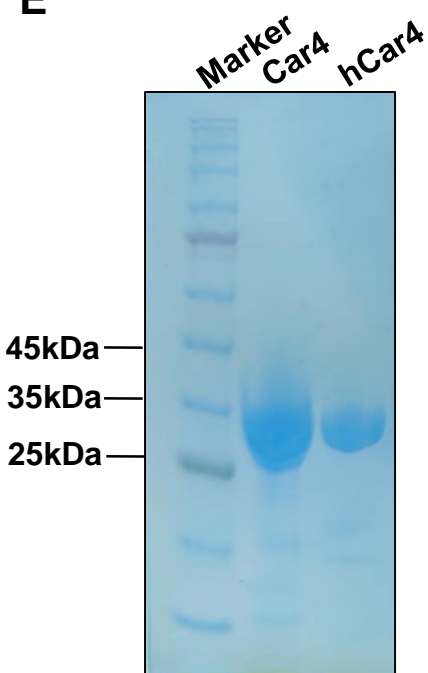**F**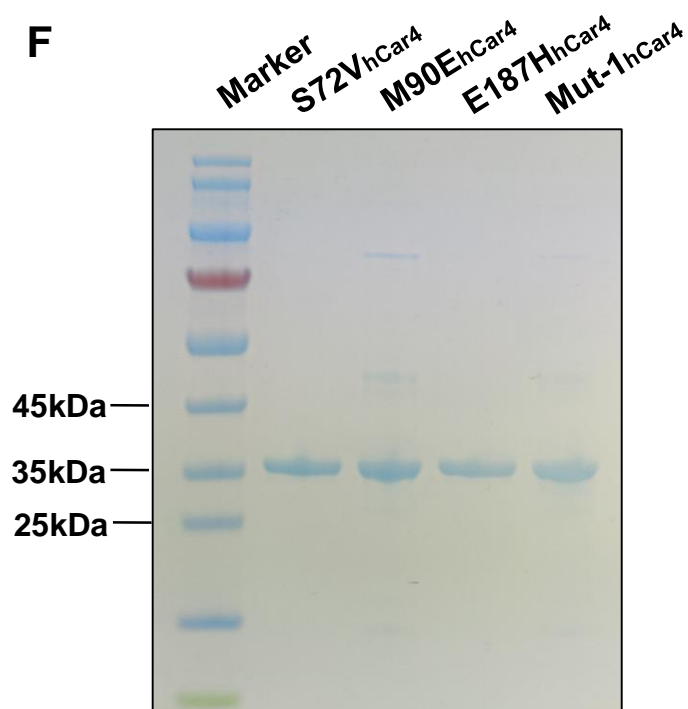

Supplement: S9 Fig — (A) The peak in gel filtration chromatography by Superdex 75 (GE). (B)-(F) SDS–PAGE of mutants of Car4, hCar4, wild-type Car4 and hCar4. The molecular weights are labeled on each panel. Car4 or hCar4 is approximately 35 kDa. (PDF) [file ppat.1011953.s009.pdf]

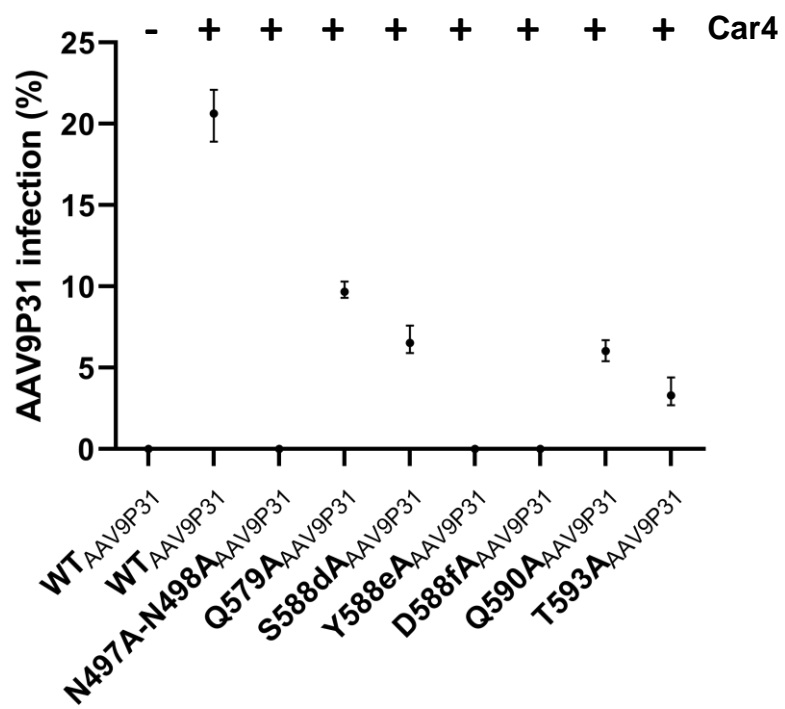

Supplement: S10 Fig — HEK293T transduced by full length Car4 were infected with mutant AAV9P31 at an MOI of 1 × 10^6^ vg cell−1. GFP expression was determined 48 h post-transduction by flow cytometry. The percentages of cells infected by AAV9P31 mutants are plotted as means ± standard errors (n = 3). (PDF) [file ppat.1011953.s010.pdf]

Car4 against AAV9

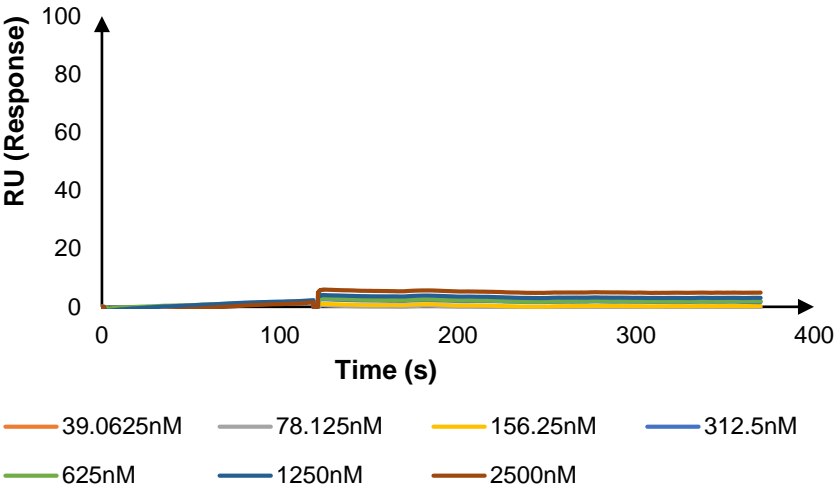

Supplement: S11 Fig — Wild-type Car4 was tested for binding capability to the AAV9 by BIAcore 8K sensorgrams. The concentrations of the analytes are indicated with different colors in each panel. RU, resonance units. (PDF) [file ppat.1011953.s011.pdf]

**A** hCar4 S72V against AAV9P31

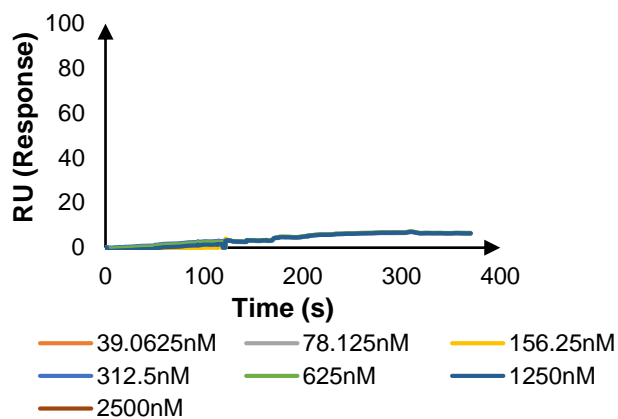

**B** hCar4 M90E against AAV9P31

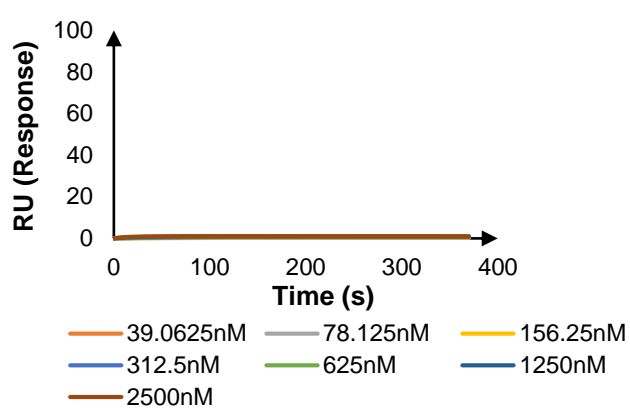

**C** hCar4 E187H against AAV9P31

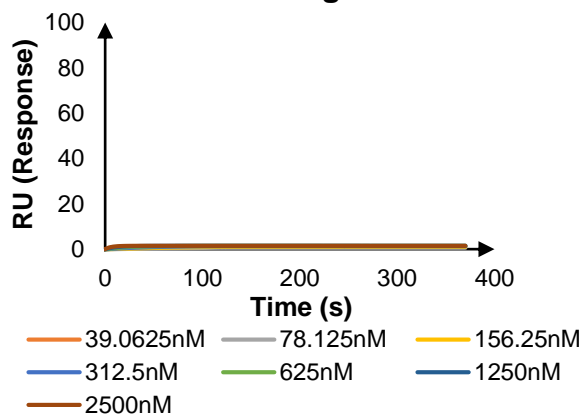

**D** hCar4 Mut-1 against AAV9P31

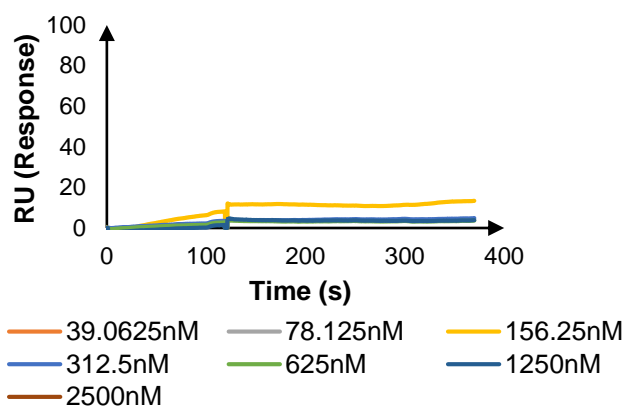

Supplement: S12 Fig — (A)-(D) Human Car4 mutants were tested for binding capability to the AAV9p31 capsid by BIAcore 8K sensorgrams. The concentrations of the analytes are indicated with different colors in each panel. RU, resonance units. (PDF) [file ppat.1011953.s012.pdf]

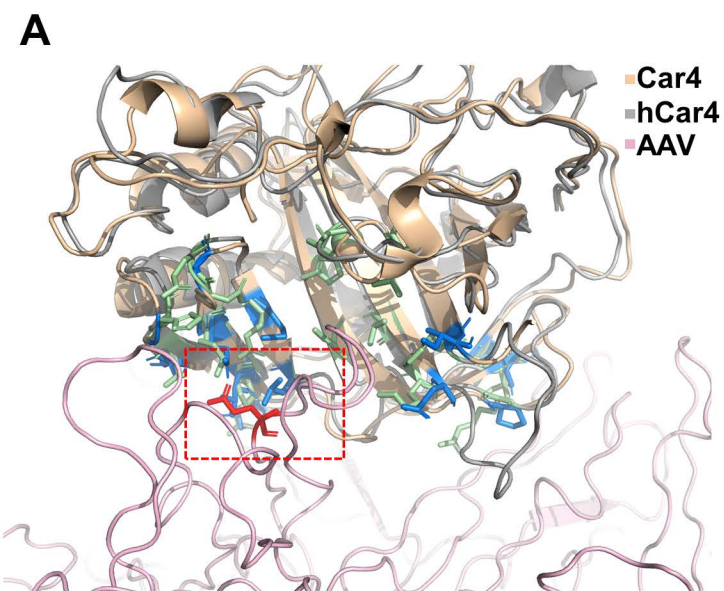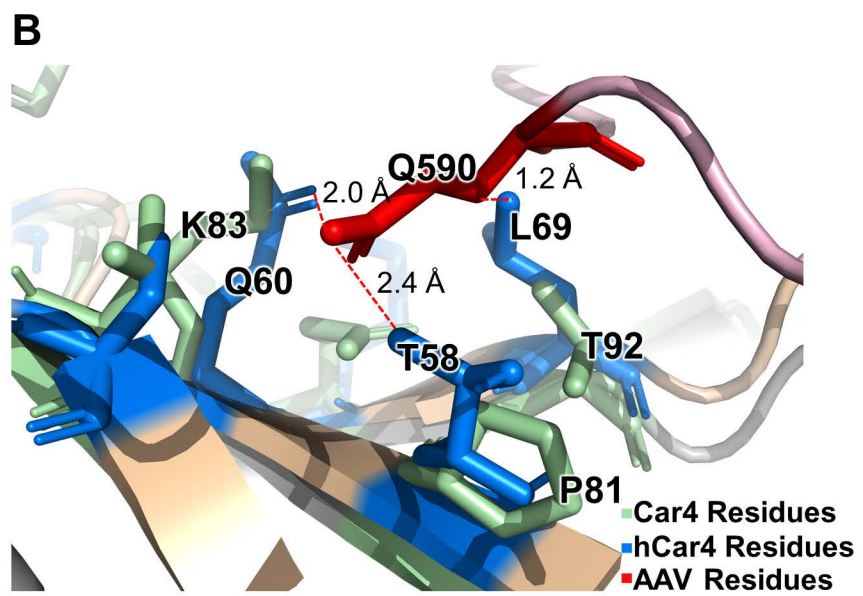

Supplement: S13 Fig — (A) hCar4 is superposed onto Car4 in the AAV9P31-Car4 complex. hCar4 is colored wheat; Car4 is colored grey; AAV9P31 virus proteins are colored pink. (B) Magnification of the red box in the left panel to highlight the details of the residues. The distances between the residues are marked. The residues of Car4 involved in the interactions are colored limon; the counterpart residues in hCar4 are colored blue; Q590AAV9P31 is colored in red. All these residues are shown as sticks. (PDF) [file ppat.1011953.s013.pdf]
