# Supplementary material for: Structural basis of the recognition of adeno-associated virus by the neurological system-related receptor carbonic anhydrase IV
Source: PLoS Pathog. 2024 Feb 5;20(2):e1011953. doi: 10.1371/journal.ppat.1011953 (PMC10868842; doi:10.1371/journal.ppat.1011953)
Supplement: S5 Fig — Overview of the cryo-EM data processing pipeline of block-based reconstruction in RELION [48,49]. (A) The I1 reconstructed 3D map of the AAV9P31-Car4 complex. (B) Blocks are isolated from the icosahedral 3-fold axes by symmetry expansion. (C) 3D classifications of 3-fold blocks. (D)-(F) Three different classes are refined in C1 symmetry. Car4 electron density presented as blue; noise presented as red. (PDF) [file ppat.1011953.s005.pdf]

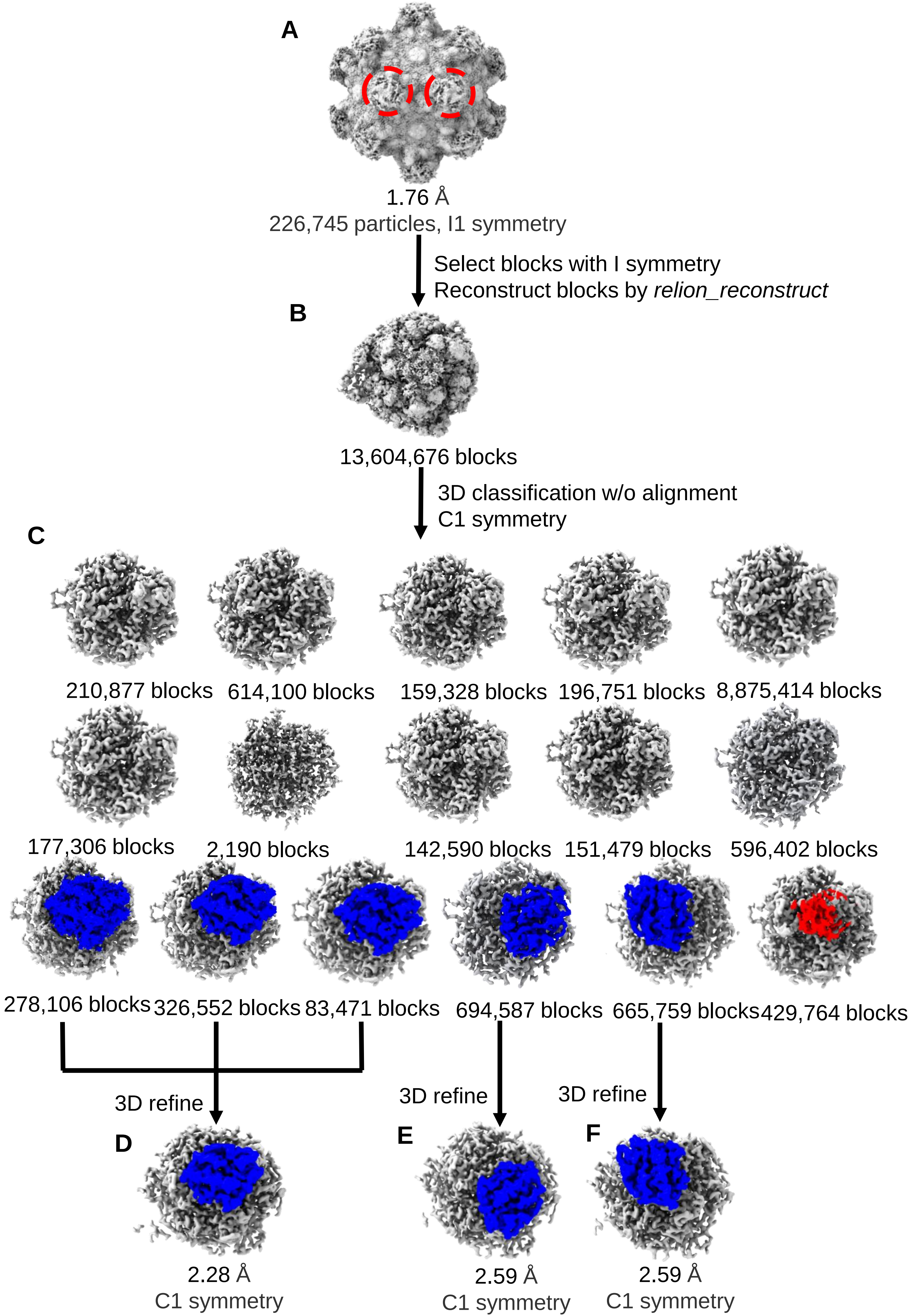
A
1.76 Å
226,745 particles, I1 symmetry
Select blocks with I symmetry
Reconstruct blocks by *relion_reconstruct*
B
13,604,676 blocks
3D classification w/o alignment
C1 symmetry
C
210,877 blocks
614,100 blocks
159,328 blocks
196,751 blocks
8,875,414 blocks
177,306 blocks
2,190 blocks
142,590 blocks
151,479 blocks
596,402 blocks
278,106 blocks
326,552 blocks
83,471 blocks
694,587 blocks
665,759 blocks
429,764 blocks
3D refine
3D refine
3D refine
D
E
F
2.28 Å
C1 symmetry
2.59 Å
C1 symmetry
2.59 Å
C1 symmetry
