# Supplementary material for: Structural basis of the recognition of adeno-associated virus by the neurological system-related receptor carbonic anhydrase IV
Source: PLoS Pathog. 2024 Feb 5;20(2):e1011953. doi: 10.1371/journal.ppat.1011953 (PMC10868842; doi:10.1371/journal.ppat.1011953)
Supplement: S1 Table — (PDF) [file ppat.1011953.s014.pdf]

	AAV9P31	AAV9P31-Car4	
**Data collection and processing**			
Magnification	105,000	105,000	
Voltage (kV)	300	300	
Electron exposure (e–/Å^2)	60	60	
Defocus range (μm)	-3.0 to -0.7	-3.0 to -0.7	
Pixel size (Å)	0.8433	0.8433	
Symmetry imposed	I1	I1	
Initial particle images (no.)	133,206	327,420	
Final particle images (no.)	116,164	226,745	
Map resolution (Å)<br>FSC threshold	1.76<br>0.143	1.76<br>0.143	
**Refined Block**			
Final particle images (no.)	/	13,604,676	
Map resolution (Å)<br>FSC threshold	/<br>/	2.28<br>0.143	
**Refinement**	AAV9P31	AAV9P31	Car4
Initial model used	AAV9	AAV9	Car4
(PDB)	7WJW	7WJW	2ZNC
Model resolution (Å)<br>FSC threshold	1.76<br>0.143	1.76<br>0.143	2.28<br>0.143
Map sharpening *B* factor (Å^2)	-53.2	-25	-10
Model composition<br>Non-hydrogen atoms<br>Protein residues<br>Ligands	 4188<br>525<br>0	 4188<br>525<br>0	 2055<br>256<br>1
B factors (Å^2)<br>Protein<br>Ligand	 59.23<br>-	 60.55<br>-	 119<br>165.03
R.m.s. deviations<br>Bond lengths (Å)<br>Bond angles (°)	 0.003<br>0.651	 0.004<br>0.737	 0.013<br>1.809
Validation<br>MolProbity score<br>Clashscore<br>Rotamers outliers (%)	 1.27<br>2.22<br>0.87	 2.08<br>5.42<br>2.62	 2.74<br>9.07<br>7.79
Ramachandran plot<br>Favored (%)<br>Allowed (%)<br>Outliers (%)	 95.98<br>3.44<br>0.57	 92.54<br>6.88<br>0.57	 88.98<br>10.63<br>0.39
