# Supplementary material for: Structural basis of the recognition of adeno-associated virus by the neurological system-related receptor carbonic anhydrase IV
Source: PLoS Pathog. 2024 Feb 5;20(2):e1011953. doi: 10.1371/journal.ppat.1011953 (PMC10868842; doi:10.1371/journal.ppat.1011953)
Supplement: S2 Table — (PDF) [file ppat.1011953.s015.pdf]

| Car4 Residues | Contacts | AAV9P31 Residues |
|---|---|---|
| Ile70 | 1,1 | Gln590, Gln592 |
| Val72 | 1 | Gln592 |
| Gln79 | 2, 1 | Gln592, Trp595 |
| Pro81 | 1, 5 | Ala591, Gln592 |
| Lys83 | 4, 1 | Gln590, Ser499 |
| Asn85 | 5 | Gln588 |
| Gln86 | 2 | Gln456 |
| Glu90 | 3 | Ala588g |
| Thr92 | 3, 8 | Ala589, Gln590 |
| Ile100 | 1 | Ala591 |
| Arg107 | 1,1 | Gln579, Thr593 |
| Gln112 | 4, 2, 1 | Trp588a, Asp588f, Ala588g |
| His114 | 1 | Tyr588e |
| Val141 | 5 | Tyr588e |
| Lys143 | 1 | Asp588f |
| Phe156 | 8, 2 | Tyr588e, Asp588f |
| His187 | 5, 8, 1 | Gly455, Gln456, Asn498 |
| Thr189 | 5, 1 | Gln590, Ser499 |
| Leu217 | 10,2 | Tyr588e, Ser588d |
| Thr219 | 6 | Ser588d |
