# Supplementary material for: Structural basis of the recognition of adeno-associated virus by the neurological system-related receptor carbonic anhydrase IV
Source: PLoS Pathog. 2024 Feb 5;20(2):e1011953. doi: 10.1371/journal.ppat.1011953 (PMC10868842; doi:10.1371/journal.ppat.1011953)
Supplement: S3 Table — (PDF) [file ppat.1011953.s016.pdf]

Ligand	Analyte	ka(1/Ms)	kd(1/s)	k_D(M)
AAV9P31	Car4	$6.30e^3$	$6.65e^{-4}$	$1.06e^{-7}$
	hCar4	-	-	-
	$I70A_{Car4}$	$1.38e^4$	$1.03e^{-2}$	$7.43e^{-7}$
	$Q79A_{Car4}$	$2.40e^4$	$7.00e^{-3}$	$2.92e^{-7}$
	$K83A_{Car4}$	-	-	-
	$T92A_{Car4}$	$6.45e^3$	$2.44e^{-3}$	$3.79e^{-7}$
	$I100A_{Car4}$	$5.61e^3$	$9.57e^{-3}$	$1.71e^{-6}$
	$R107A_{Car4}$	$7.09e^3$	$4.48e^{-3}$	$6.31e^{-7}$
	$E109A_{Car4}$	-	-	-
	$Q112A_{Car4}$	-	-	-
	$V141A_{Car4}$	-	-	-
	$H187A_{Car4}$	-	-	-
	$L217A_{Car4}$	-	-	-
	$T219A_{Car4}$	$9.27e^3$	$1.28e^{-2}$	$1.39e^{-6}$
	$D223A_{Car4}$	$1.66e^4$	$1.15e^{-3}$	$6.92e^{-8}$
	$V72S_{Car4}$	$1.05e^4$	$6.35e^{-4}$	$6.07e^{-8}$
	$E90M_{Car4}$	$5.37e^3$	$1.74e^{-4}$	$3.23e^{-8}$
	$H187E_{Car4}$	$1.70e^4$	$3.49e^{-6}$	$2.06e^{-10}$
	Mut-1_{Car4}	$4.08e^4$	$3.55e^{-4}$	$8.71e^{-9}$
	Mut-2_{hCar4}	$8.22e^3$	$3.77e^{-4}$	$4.59e^{-8}$
	$S72V_{hCar4}$	-	-	-
	$M90E_{hCar4}$	-	-	-
	$E187H_{hCar4}$	-	-	-
	Mut-1_{hCar4}	-	-	-
AAV9	Car4	-	-	-
